# Ni Single‐Atom Modulation of Ti‐O Covalency Boosts Ammonia Oxidation Electrocatalysis

**DOI:** 10.1002/advs.202521932

**Published:** 2026-01-20

**Authors:** Subhash Chandra Shit, Dayoung Kwon, Nhi Thi Yen Phan, Hyo Won Kim, Jucheol Park, Jeong‐hyeon Lee, Hyeyoung Shin, Wooyul Kim

**Affiliations:** ^1^ Department of Energy Engineering Korea Institute of Energy Technology (KENTECH) Naju Republic of Korea; ^2^ Graduate School of Energy Science and Technology (GEST) Chungnam National University Daejeon Republic of Korea; ^3^ Center for Shared Research Facilities Korea Institute of Energy Technology (KENTECH) Naju Republic of Korea

**Keywords:** ammonia oxidation reaction, covalency modulation, in situ spectroscopy, metal–support interaction, single‐atom catalysts (SACs)

## Abstract

Non‐noble transition metal oxides, particularly TiO_2_‐based systems, can be an alternative to noble metal‐based catalysts for the electrochemical ammonia oxidation reaction (AOR) due to their abundance, low cost, and corrosion resistance, but it remains hindered by lower performance and undesired selectivity toward oxygenated nitrogen species instead of N_2_, largely stemming from insufficient active sites and higher energy barrier for coupling intermediates. To overcome the issues, we introduce a Ni single‐atom‐induced covalent modulation strategy for constructing Ni SAC@TiO_2_ with tunable Ti‐O covalency. Hard and soft X‐ray absorption (XAS) combined with photoelectron spectroscopy (XPS) reveal strong metal‐support interactions that enhance Ti‐O covalency and create abundant active sites. Ni SAC@TiO_2_ catalyst nearly doubles the catalytic activity of pristine TiO_2_ and retains >98% of its initial performance after 2000 accelerated stress testing cycles. In situ surface‐enhanced Raman scattering (SERS) shows improved interaction with reactants and intermediates, while in situ attenuated total reflection‐surface enhanced infrared absorption spectroscopy (ATR‐SEIRAS) demonstrates that Ni SAC@TiO_2_ effectively suppresses the buildup of deactivating NO*_x_* species and promotes NH*_x_*‐NH*_y_* coupling mediated pathways for selective N_2_ evolution, further corroborating the theoretical insights. These findings highlight single‐atom modulation of Ti‐O covalency as a powerful strategy to unlock efficient and robust TiO_2_‐based catalysts for AOR.

## Graphical abstract

A Ni single‐atom‐induced enhancement of Ti‐O covalency in Ni SAC@TiO_2_ generates abundant active sites and promotes selective NH*_x_*‐NH*_y_* mediated N_2_ evolution while suppressing poisonous NO*_x_* formation, achieving nearly doubled AOR activity with excellent durability.

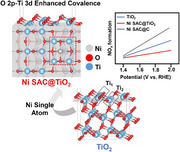

## 1 Introduction

Ammonia electrooxidation (AOR) has emerged as a promising pathway for sustainable hydrogen production, next‐generation fuel cells, and advanced wastewater treatment [1, 2, 3, 4]. Noble metals such as Pt, Ir, and Rh exhibit excellent AOR performance with low overpotentials (∼0.6 V) but are limited by high cost, scarcity, and susceptibility to nitrogen (N) adatom poisoning, which restricts their large‐scale deployment [5, 6, 7, 8, 9]. In contrast, coinage metals such as Au, Ag, and Cu display intrinsically low AOR activity due to weak nitrogen adsorption and limited capability to dehydrogenate ammonia into key intermediates (NxHy) [10]. Among non‐precious alternatives, nickel‐based catalysts have gained attention for their strong nitrogen binding and competitive AOR performance; however, their practical viability remains limited by poor long‐term durability, as continuous nickel leaching under alkaline conditions and unfavorable oxidation pathways that preferentially yield oxygenated nitrogen species rather than molecular nitrogen (N_2_) pose critical challenges for stable AOR operation [11, 12].

Recently, non‐noble metal oxides have emerged as promising alternatives for electrochemical oxidation reactions [13, 14, 15, 16, 17, 18], including the AOR [19]. Motivated by the tunable electronic structure of perovskite oxides, Tao and co‐workers developed the ABO_3_‐type LNCO55‐Ar system, revealing that annealing under an Ar atmosphere, in combination with B‐site cation substitution, effectively generates a high concentration of oxygen vacancies that act as active sites for AOR [20]. Building on this concept, K_2_NiF_4_‐type perovskite oxides with dual‐site cation substitution have been explored to further boost catalytic activity while suppressing the formation of heterogeneous secondary phases, thereby achieving enhanced performance [21]. Similarly, introducing oxygen vacancies can activate otherwise inactive metal oxides; for example, defect engineering has rendered CuO catalytically active for AOR [22]. Building on this concept, Chen and co‐workers created vacancy defects in NiCuO*_x_* bimetallic catalysts via simple hydrogenation, which exposed additional adsorption‐active sites favorable for AOR [23]. In parallel, titanium‐based oxides, known for their low cost and excellent corrosion resistance, have also attracted attention as potential AOR electrocatalysts. For instance, Zhao and co‐workers synthesized defect‐rich TiO nanofibers through hydrogenation of TiO_2_, where they assumed introduced oxygen vacancies might act as active sites for AOR [24]. Nevertheless, under alkaline aqueous conditions, Ti‐based oxides face sluggish reaction kinetics, resulting in low overall activity and a tendency to produce oxygenated nitrogen species rather than molecular nitrogen (N_2_). Overall, the AOR performances of non‐noble‐metal oxide catalysts are still far less than those of cutting‐edge platinum‐based systems [10]. Given their high abundance, low cost, and inherent corrosion resistance, TiO_2_‐based electrocatalysts warrant deeper investigation. The central challenge is to precisely control active sites, such as oxygen vacancies, and tailor Ti─O bonding to enhance activity. At the same time, a clearer understanding of reactant–surface interactions and the underlying AOR mechanism on Ti‐based catalysts is essential to suppress nitrogen oxide by‐products and enable selective N_2_ generation. A key avenue for improving the performance of metal oxides lies in the rational tuning of metal‐oxygen (M‐O) interactions within their lattice and the controlled generation of vacancy sites [25]. Strengthening M‐O covalency has been shown to enhance d‐electron delocalization, stabilize high‐spin metal centers, and optimize intermediate adsorption‐critical factors for lowering reaction barriers. For example, Grimaud et al. identified M‐O covalency as a pivotal descriptor for oxygen evolution reaction (OER) activity, although excessive covalency can trigger lattice oxygen mobility and thus undermine catalyst stability [26]. Achieving an optimal balance between covalency and ionicity, therefore, remains a central challenge in designing high‐performance metal oxide catalysts. [27] Recent advances in single‐atom catalysts (SACs) offer a powerful platform to achieve this balance. Through strong metal–support interactions (SMSI), SACs can precisely tune M–O covalency while stabilizing isolated active sites, enabling the support itself to participate in catalysis [28]. This approach provides a compelling strategy to design TiO_2_‐based catalysts that combine activity and durability for efficient AOR.

Building on these insights, we report a Ni single‐atom‐induced covalent modulation strategy for constructing Ni SAC@TiO_2_ with tunable Ti─O covalency. Ni SAC@TiO_2_ exhibits markedly enhanced catalytic activity and operational stability compared with pristine TiO_2_, as well as with control systems including Cu SAC@TiO_2_, Co SAC@TiO_2_, Ni SAC@C, and Ni NP@TiO_2_. To elucidate the nature of the active sites, we performed a comprehensive characterization using both hard and soft XAS studies. The mechanistic pathways and catalyst‐reactant interactions responsible for the enhanced AOR performance were further elucidated using in situ SERS and in situ ATR‐SEIRAS analysis. Computational investigations on the mechanism, charge density difference, and electronic properties combined with experimental observations confirmed the enhanced AOR activity of the Ni SAC@TiO_2_ catalyst.

## 2 Results and Discussion

### 2.1 Structure and Electronic Interaction: Electron Microscopy and X‐Ray Techniques including XPS, XANES, and EXAFS

Ni single‐atom catalysts supported on TiO_2_ (Ni SAC@TiO_2_) were synthesized using a modified wrap‐bake‐peel strategy, which enables effective stabilization of isolated metal atoms on the oxide support. [29] Following a similar procedure, Cu SAC@TiO_2_ and Co SAC@TiO_2_ were also prepared to allow comparative evaluation of different transition‐metal centers. For additional benchmarking, Ni SAC supported on carbon (Ni SAC@C) was obtained through incipient wet impregnation followed by calcination [30], while bare TiO_2_ and Ni nanoparticles supported on TiO_2_ (Ni NP@TiO_2_) were synthesized via a conventional sol‐gel method [31] as control samples (See Section 4 for details). ICP‐AES was employed to quantify the metal loadings in all catalysts (Table S1). The SAC systems exhibited nearly identical and well‐controlled loadings, ensuring that observed performance trends arise primarily from differences in atomic dispersion and support effects rather than metal content.

**FIGURE 1:**
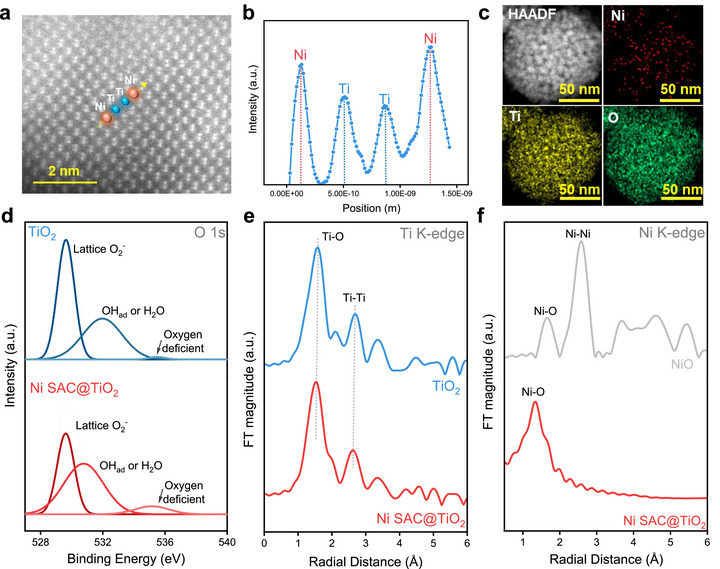
Structural and electronic characterization of Ni SAC@TiO_2_ and TiO_2_. (a) HAADF‐STEM image of Ni SAC@TiO_2_ with Ti (cyan) and Ni (red) atom positions. (b) Line‐scan intensity profile confirming atomically dispersed Ni. (c) STEM‐EDS elemental mapping showing uniform Ni, Ti, and O distribution. (d) O 1s XPS spectra revealing surface oxygen modulation upon Ni incorporation. (e) Fourier‐transformed EXAFS spectra at the Ti K‐edge showing shifts in Ti─O and Ti─Ti coordination after Ni loading. (f) Ni K‐edge EXAFS spectra confirming single‐atom Ni without Ni─Ni coordination.

First, HAADF‐STEM was used to visualize the isolated Ni atoms in the Ni SAC@TiO_2_ catalyst. Due to the higher atomic number of Ni relative to Ti, individual Ni atoms appear as brighter contrast spots distinctly aligned along the Ti atomic rows (Figure 1a), as further confirmed by line scan analysis (Figure 1b). To unequivocally distinguish Ni atoms from Ti and to rule out imaging artefacts, we complemented STEM imaging with atomic‐resolution EELS analysis. The EELS spectra (Figure S1) clearly exhibit the characteristic Ni L_3_ and L_2_ edges, confirming the chemical identity of the bright atomic features observed in HAADF‐STEM. The spatially resolved EELS maps further validate that Ni exists exclusively in an isolated, atomically dispersed form. Similar analyses were performed for the Cu SAC@TiO_2_ and Co SAC@TiO_2_ control catalysts, where bright atomic spots in HAADF‐STEM (Figures S2 and S3) and their corresponding Cu/Co L‐edge signals in the EELS spectra verified the successful formation of Cu and Co single‐atom sites on TiO_2_ [32]. Furthermore, STEM‐EDS elemental mapping was conducted to assess the elemental distribution within each catalyst. For Ni SAC@TiO_2_ (Figure 1c), the EDS maps show a uniform, homogeneous dispersion of Ni across the TiO_2_ support with no signs of local enrichment or aggregation, consistent with a single‐atom distribution. Control samples also exhibited expected elemental signatures: Ti and O in pristine TiO_2_; Cu, Ti, and O in Cu SAC@TiO_2_; Co, Ti, and O in Co SAC@TiO_2_; Ni, Ti, and O in Ni NP@TiO_2_; and Ni, C, and O in Ni SAC@C (Figures S4–S8). No extraneous elemental impurities were detected in any sample. Low‐ and high‐resolution TEM analyses (Figures S9–S12) further confirmed the morphological and crystalline features of the catalysts. All TiO_2_‐based materials‐TiO_2_, Ni SAC@TiO_2_, and Ni NP@TiO_2_‐consist of spherical TiO_2_ nanocrystals with clearly resolved lattice fringes corresponding to both anatase and rutile phases. Notably, the anatase (101) plane with a d‐spacing of 0.346 nm [33] was dominantly exposed in Ni SAC@TiO_2_, suggesting preferential surface stabilization facilitated by Ni single atoms. In the Ni NP@TiO_2_ sample, no crystalline lattice fringes corresponding to Ni or NiO were observed, indicating that the Ni nanoparticles are primarily amorphous. In contrast, high‐resolution TEM images of Ni SAC@C revealed onion‐like carbon nanoparticles with well‐defined graphitic lattice fringes [34, 35].

Powder X‐ray diffraction (PXRD) patterns of TiO_2_, Ni SAC@TiO_2_, Cu SAC@TiO_2_, Co SAC@TiO_2_, and Ni NP@TiO_2_ (Figure S13) confirm that all samples exhibit a mixed‐phase TiO_2_ structure comprising characteristic reflections of both anatase and rutile, consistent with the lattice fringes observed in the HRTEM images. A notable feature of the Ni SAC@TiO_2_ pattern is the pronounced enhancement of the anatase (101) peak intensity upon incorporation of isolated Ni atoms. Unlike Ni NP@TiO_2_, which exhibits a PXRD pattern nearly identical to pristine TiO_2_, the Ni SAC@TiO_2_ sample shows a distinctly sharper and more intense (101) reflection. This phenomenon is attributed to the strong electronic interactions between atomically dispersed Ni centers and the TiO_2_ lattice, which induces slight lattice ordering and reduces defect‐related broadening, resulting in a sharper and more intense (101) reflection. Additionally, single‐atom anchoring preferentially stabilizes the surface oxygen sublattice, promoting improved crystallinity along the (101) direction, which aligns well with the HRTEM observations of well‐resolved lattice fringes. Importantly, no diffraction peaks corresponding to Ni, NiO, or Ni_2_O_3_ were detected in Ni SAC@TiO_2_, confirming that Ni remains in an atomically dispersed state without forming crystalline nanoparticles. Similarly, the PXRD patterns of Cu SAC@TiO_2_ and Co SAC@TiO_2_ show no detectable peaks related to Cu/CuO/Cu_2_O or Co/Co_3_O_4_, indicating that both Cu and Co species are also present as isolated single atoms anchored on TiO_2_. This is fully consistent with the HAADF‐STEM and EELS analyses, which reveal exclusively single‐atom features for these catalysts [32]. In contrast, the Ni NP@TiO_2_ sample shows no crystalline signatures of metallic Ni or NiO phases, despite the presence of Ni nanoparticles. The absence of Ni‐related reflections suggests that the Ni species in this sample are predominantly amorphous, as corroborated by the absence of Ni/NiO lattice fringes in the corresponding high‐resolution TEM images [31].

To explore the surface valence state and elemental composition of the catalysts, XPS measurements were conducted (Figure 1d; Figures S14–S17). In the Ti 2p spectra, two peaks were identified at 458.38 and 464.38 eV, as Ti 2p_3/2_ and Ti 2p_1/2_, respectively [36], confirming the presence of oxidized TiO_2_ in both TiO_2_ and Ni SAC@TiO_2_. The O 1s peak in both samples could be resolved into three peaks, representing lattice oxygen [36], oxygen bound to Ti atoms in hydroxyl groups, and oxygen in a deficient environment due to Ni interaction with TiO_2_ [37], with the Ni SAC@TiO_2_ sample showing a relatively higher binding energy due to strong Ni‐TiO_2_ interaction, resulting in a higher oxygen vacancy. For Ni SAC@C, only one peak was observed in the O 1s XPS spectrum, associated with oxygen bound to hydroxyl groups adsorbed by Ni atoms. Confirmation of Ni SAC was achieved by analyzing by Ni 2p XPS analysis, which exhibited characteristic peaks at 855.0 eV and 872.5 eV, corresponding to Ni 2p_3/2_ and Ni 2p_1/2_, respectively. The binding energy of Ni 2p_3/2_, positioned lower than that of Ni^2+^ yet higher than that of metallic Ni, indicates that the Ni species in Ni SAC@TiO_2_ possess a low‐valence state between 0 and +2 [38].

To obtain a deeper understanding of the electronic structure and local coordination environment, XAS was conducted, comprising both XANES and EXAFS analyses. (Figure 1e,f; Figures S18–S21). The XANES spectra at the Ti K‐edge (Figure S18a) for Ni SAC@TiO_2_ samples closely resemble those of bare TiO_2_. A modest increase in the pre‐edge intensity is observed for both Ni SAC@TiO_2_ and bare TiO_2_, which can be attributed to the presence of more distorted TiO_6_ octahedra and an increase in structural defects. This enhancement in pre‐edge intensity suggests that the introduction of Ni leads to increased asymmetry in the Ti local structure. Furthermore, the reduced intensity of the main absorption peak for Ti K‐edge in Ni SAC@TiO_2_ compared to pure TiO_2_ implies a partial reduction of Ti species, which is likely resulting from charge compensation or interactions between Ni and the TiO_2_ matrix [39].

The EXAFS analysis with fitting of the Ti K‐edge (Figure 1e; Figures S19a,b and S20a,b) provides further insight into the structural changes induced by the introduction of Ni SAC. The Fourier‐transformed (FT) k^3^‐weighted EXAFS spectra for TiO_2_ exhibit two prominent peaks at 1.57 and 2.69 Å, corresponding to the Ti─O and Ti─Ti bond distances, respectively [40]. These peaks represent the first‐shell coordination of oxygen atoms around Ti and the second‐shell coordination involving neighboring Ti atoms in the TiO_2_ lattice. In the case of Ni SAC@TiO_2_, a noticeable contraction in the Ti─O bond length is observed, shifting from 1.57 Å in undoped TiO_2_ to 1.52 Å (Table S2). This contraction is accompanied by a broader peak distribution, indicative of greater variability in the local Ti─O bond environment. Such changes suggest the formation of a higher concentration of surface defects and lattice distortions, likely arising from the interaction between Ni single atoms and the TiO_2_ support [39]. These defect sites may enhance the reactivity of the material by increasing the availability of active sites for catalytic processes. Overall, the EXAFS findings corroborate the XANES results, providing a comprehensive picture of the structural modifications in Ni SAC@TiO_2_. The presence of contracted Ti─O bonds and defect‐rich regions highlights the significant impact of Ni SAC on the TiO_2_ lattice, offering insights into the material's enhanced electrocatalytic AOR performance.

The XANES spectra at the Ni K‐edge (Figure S18b) and its derivative spectra (Figure S18c) provide key insights into the local electronic structure and coordination environment of Ni atoms in Ni‐SAC@TiO_2_. Unlike NiO, the Ni‐SAC exhibits significant differences in its local atomic structure, as evidenced by variations in the absorption edge which reveal intermediate oxidation states for Ni in Ni‐SAC, distinguishing it from the fully oxidized state (+2) typically observed in NiO. Enhanced pre‐edge features and shoulder peaks, attributed to 1s→3d and 1s→4pz transitions, further suggest that the Ni atoms in Ni SAC@TiO_2_ and Ni‐SAC@C adopt a planar coordination geometry rather than the octahedral structure found in NiO. The Ni K‐edge absorption energy of Ni‐SAC@TiO_2_ and Ni SAC@C are slightly lower than that of NiO, indicating that the valence state of Ni species is close to +2 but not fully equivalent to it, consistent with XPS analysis [41, 42]. To further elucidate the bonding and coordination environment of Ni in the SACs, Ni K‐edge EXAFS analysis was performed, and the Fourier‐transformed (FT) spectra with fitting are shown in Figure 1f and Figures S19c,d, S20c,d, and Table S3. The EXAFS spectrum of Ni‐SAC@TiO_2_ reveals a prominent peak at 1.35 Å, corresponding to the Ni─O coordination. This bond length is shorter than the Ni─O distance in bulk NiO (1.65 Å), indicating a distorted local environment for Ni atoms in Ni‐SAC@TiO_2_. The absence of a Ni‐Ni coordination peak at 2.52 Å confirms the atomic dispersion of Ni, with no evidence of Ni nanoparticles or clusters [43, 44]. These findings are consistent with other structural characterizations, such as HAADF‐STEM and XRD, which also support the single‐atom nature of Ni in the catalyst. Additionally, EXAFS analysis of other SACs, such as Ni‐SAC@C (Figure S21), reveals distinct features in the coordination environment. Ni‐SAC@C exhibits a primary peak at 2.22 Å, which is attributed to Ni─C bonding. This is markedly different from the peaks observed for Ni─O (1.65 Å) and Ni─Ni (2.58 Å), further underscoring the variability in coordination geometry depending on the support material [43]. The EXAFS results provide compelling evidence of atomic‐level dispersion of Ni atoms in Ni‐SAC@TiO_2_, with unique bonding and coordination environments distinct from bulk NiO.

### 2.2 Enhancing Ti─O Covalency: Soft X‐Ray Absorption Spectroscopy

**FIGURE 2:**
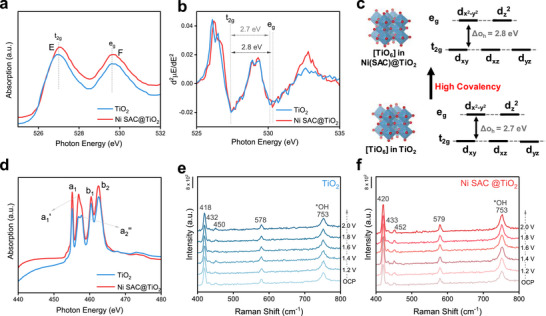
Electronic structure, catalyst interaction, and in situ SERS analysis of TiO_2_ and Ni SAC@TiO_2_. (a) O K‐edge soft XAS spectra (524–532 eV), (b) corresponding second‐derivative plots, and (c) comparison of octahedral (O_h_) crystal‐field splitting, highlighting enhanced Ti─O covalency in Ni SAC@TiO_2_. (d) Ti L‐edge soft XAS spectra of TiO_2_ and Ni SAC@TiO_2_. In situ SERS analysis recorded under varying oxidation potentials in 0.1 m NaClO_4_ with 0.1 m NH_3_ for (e) TiO_2_ and (f) Ni SAC@TiO_2_.

To enhance our comprehension of the electronic parameter, proper phase and oxidation state, we conducted soft XAS analyses on all catalysts (Figure 2; Figures S22 and S23). The Ni L‐edge displays two distinct groups of peaks around 845–860 eV (L_3_‐edge) and 865–876 eV (L_2_‐edge), attributed to the splitting of the Ni 2p orbitals (Figure S22a). The peaks observed for Ni NP closely align with the NiO peak, indicating the presence of amorphous NiO in Ni NP@TiO_2_. Notably, there exists a discernible difference in peak position between Ni NP and Ni SAC, suggesting distinct coordination environments [45]. While Ni SAC demonstrates a relatively broad peak, signifying a more complex coordination environment for the single Ni atoms, the peak intensity for Ni SAC@TiO_2_ surpasses that of Ni SAC@C [46]. This discrepancy could be attributed to the heightened interaction between Ni SAC and TiO_2_. Consequently, this interaction contributes to the superior cyclic stability observed in the electrochemical AOR of Ni SAC@TiO_2_ compared to Ni SAC@C. The C K‐edge XAS spectrum of the Ni SAC@C sample displays four distinct absorption peaks (Figure S22b), which provide valuable information regarding the carbon support framework. The first peak at 284.7 eV corresponds to the 1s → π^*^ transition associated with C═C bonds, while the second peak at 286.9 eV is attributed to the 1s → π^*^ transition of C═O and C─OH functional groups. A third feature at 287.6 eV originates from the 1s → σ^*^ transition involving aliphatic moieties such as ─CH─, ─CH_2_─, and ─CH_3_. Finally, the broad peak observed at 292.1 eV arises from the delocalized π states of the graphitic network [47].

The O K‐edge XAS spectra of the TiO_2_‐based samples exhibit characteristic features comprising two well‐defined sub‐bands near the absorption threshold (525–535 eV), designated as E and F, along with a broader region at higher energies (535–550 eV), labeled G, H, and I (Figure 2a; Figure S23a). The low‐energy doublet originates from the hybridization between O 2p and Ti 3d orbitals, resulting in the formation of t_2g_ and e_g_ sub‐bands (see derivative spectra in Figure 2b). The energy separation between these bands reflects the optical crystal field splitting (ΔCF), which is sensitive to variations in the local coordination and ligand environment. The higher‐energy region (G–I) corresponds to transitions involving O 2p‐Ti 4sp orbital hybridization, reflecting the long‐range ordering in TiO_2_. Typically, the E‐F splitting for TiO_2_ is around 2.7 eV; in our study, this value was found to be approximately 2.8 eV, with the e_g_ peak slightly shifted to higher energy, suggesting enhanced Ti‐O covalency and strong Ni─TiO_2_ interfacial interactions (Figure 2c). Moreover, the E and F peaks in Ni SAC@TiO_2_ appear at higher energies compared to Ni NP@TiO_2_, further supporting stronger coupling between Ni single atoms and the TiO_2_ support. In addition, three broad high‐energy features, denoted as G, H, and I, are primarily associated with oxygen‐derived states. Peaks G and H correspond to O 1s → t_1u_ transitions to the lowest unoccupied molecular orbitals, while peak I arises from O 1s → t_1u_ transitions to higher unoccupied states. Among all samples, the Ni SAC@TiO_2_ system exhibits the most intense signals, indicative of stronger metal‐support interactions and enhanced structural stability [48, 49].

The Ti L‐edge XAS spectra (Figure 2d; Figure S23b) display two prominent sets of sharp resonances, labeled a_1_, a_2,_ and b_1_, b_2_, corresponding to the L_3_ and L_2_ edges, respectively. The fine splitting within each edge arises due to spin–orbit coupling and crystal‐field effects. The relative intensity and energy separation between the L_3_ doublet (a_1_/a_2_) provide an estimate of the crystal field strength (10 Dq)‐a larger field strength manifests as a more intense a_1_ peak and a wider energy gap between a_1_ and a_2_. In our case, the Ni SAC@TiO_2_ sample demonstrates a stronger crystal field than Ni NP@TiO_2_, implying a more robust metal‐support interaction. Furthermore, tetragonal distortion in TiO_2_ alters the local Ti site symmetry to approximately D_4h_ (rutile) or D_2d_ (anatase), leading to the further splitting of the L_3_‐edge second peak (a_2_) into two components, a_2_′ and a_2_″. The relative intensity relationship between these sub‐peaks serves as a phase indicator: a_2_′ > a_2_″ is typical of anatase, while a_2_′ < a_2_″ corresponds to rutile. In this work, Ni SAC@TiO_2_ exhibits a_2_′ > a_2_″, consistent with the dominance of the anatase phase, whereas Ni NP@TiO_2_ shows a_2_′ < a_2_″, signifying a rutile‐rich structure [50]. These observations are consistent with the findings from powder X‐ray diffraction (PXRD).

### 2.3 Electrochemical Ammonia Oxidation Reaction (AOR) Performance

**FIGURE 3:**
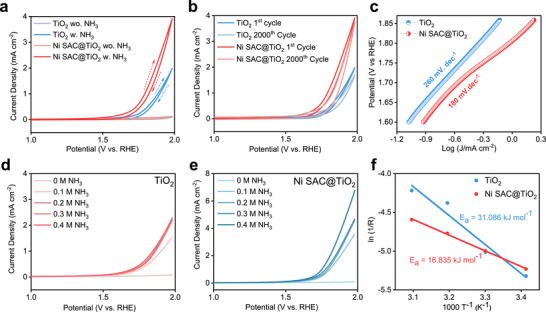
Electrochemical AOR performance of TiO_2_ and Ni SAC@TiO_2_. (a) CV profiles in Ar‐saturated 0.1 m NaClO_4_ with and without 0.1 m NH_3_ within 1–2 V vs. RHE at 10 mV s^−1^. (b) AST comparing AOR stability of TiO_2_ and Ni SAC@TiO_2_; darker and lighter lines represent the 1st and 2000th cycles. (c) Tafel plots derived from LSV data, showing enhanced AOR kinetics of Ni SAC@TiO_2_. (d, e) LSV curves of TiO_2_ (d) and Ni SAC@TiO_2_ (e) at varying NH_3_ concentrations. (f) Apparent activation energies determined from temperature‐dependent EIS measurements in the same electrolyte.

To evaluate the electrochemical AOR activity relative to the competing oxygen evolution reaction (OER), the catalysts were systematically tested in Ar‐saturated 0.1 m NaClO_4_ electrolyte, both with and without 0.1 m aqueous NH_3_, using a conventional three‐electrode setup with a Pt wire counter electrode and an Ag/AgCl reference electrode. The corresponding cyclic voltammograms (CVs) and linear sweep voltammetry (LSV) curves recorded in the potential range of 0–2 V vs. RHE are presented in Figure 3 and Figures S24 and S25. As shown in Figure 3a, the reaction potential of the Ni SAC@TiO_2_ and TiO_2_ is 1.80 and 1.90 V vs. RHE, respectively, to drive the current density of 1 mA cm^−2^ where there is almost negligible OER current density at that potential. On the other hand, Cu and Co SAC@TiO_2_ (Figure S24) display AOR activity similar to that of bare TiO_2_, while Ni SAC@TiO_2_ achieves peak current densities approximately 2.4 times higher, highlighting the unique role of Ni single‐atom incorporation in enhancing AOR activity. Furthermore, LSV measurements in electrolytes with varying NH_4_OH concentrations (Figure 3d,e) reveal a clear ammonia‐concentration‐dependent enhancement in AOR activity for Ni SAC@TiO_2_. In comparison, TiO_2_ shows a slight increase in current density when the NH_4_OH concentration increases from 0.1 to 0.2 m but reaches a plateau at higher concentrations (0.3–0.4 m), indicating limited capability for further activity gains. These results suggest that the incorporation of Ni single atoms effectively tunes the electronic structure of TiO_2_ [51], improving its ammonia adsorption‐desorption dynamics and facilitating more efficient oxidation pathways. To rule out the contribution of metallic Ni nanoparticles, we tested a Ni nanoparticle‐decorated TiO_2_ (Ni NP@TiO_2_) control sample. Unlike the Ni SAC@TiO_2_, the Ni NP@TiO_2_ exhibited distinct redox peaks (∼0.5–1.0 V vs. RHE) corresponding to the Ni^2+^/Ni^3+^ redox transition (Figure S25b) [52], which are absent in the Ni SAC@TiO_2_ system, confirming the atomic dispersion of Ni in the latter and its unique catalytic behavior. Furthermore, we have performed comprehensive product analyses to unambiguously identify the reaction products and evaluate their Faradaic efficiencies (FEs) (Figures S26 and S27 and Table S4). Specifically, the gaseous products were analyzed using gas chromatography (GC), while the liquid‐phase products were quantified by UV–vis spectroscopy. These combined analyses allowed us to determine the FEs toward N_2_, O_2_, and nitrate (NO_3_^−^) for all catalysts, including Ni SAC@TiO_2_, TiO_2_, Cu SAC@TiO_2_, and Co SAC@TiO_2_. These results clearly demonstrate that Ni SAC@TiO_2_ exhibits the highest selectivity toward N_2_ and significantly suppressed production of NO_3_^−^, in stark contrast to pristine TiO_2_ and the Cu and Co SACs, all of which generate substantial amounts of nitrate. The correlation between product selectivity and electrochemical activity confirms that TiO_2_, Cu SAC, and Co SAC suffer from stronger poisoning and less effective NH_3_ activation, whereas Ni SAC@TiO_2_ promotes the desired N_2_ pathway by minimizing nitrogen oxides intermediates. Additionally, we have included a comprehensive comparative table (Table S5) that summarizes key performance metrics, such as onset potential and current density, for the present Ni SAC@TiO_2_ catalyst alongside state‐of‐the‐art non‐noble metal‐based NH_3_ oxidation electrocatalysts reported in the literature. The quantitative comparison shows that, although some bimetallic or trimetallic systems exhibit lower onset potentials or higher current densities, the Ni SAC@TiO_2_ catalyst achieves competitive performance despite using a single‐metal design with an ultralow Ni loading (0.302 wt.%) and lower NH_3_ concentration mainly due to the strong metal–support interaction and enhanced covalency.

To evaluate the stability of the AOR performance, chronoamperometry (CA) and accelerated stress testing (AST) cycles were conducted. In the CA experiments performed at ∼1.7 V vs. RHE, both catalysts exhibited an initial rapid decay in current density, attributed to the adsorption of nitrogen species on the catalyst surface (Figure S28). Notably, Ni SAC@TiO_2_ maintained a higher steady‐state current than TiO_2_ throughout the test. To further assess durability, AST cycles were carried out of 2000 continuous CV cycles within a potential window of 0–2 V vs. RHE. As shown in Figure 3b, the peak current density of TiO_2_ decreased by ∼20% after cycling, likely due to NO*_x_* adsorption blocking active sites [17], whereas Ni SAC@TiO_2_ retained nearly full activity. Control catalysts, Ni NP@TiO_2_ and Ni SAC@C (Figure S25a,b), initially showed lower onset potentials (1.69 and 1.76 V vs. RHE, respectively) but suffered rapid degradation. Ni NP@TiO_2_ underwent nanoparticle leaching, evidenced by the disappearance of the Ni^2+^/Ni^3+^ redox peak and convergence of its activity to that of bare TiO_2_. Ni SAC@C was also severely deactivated. In contrast, Ni SAC@TiO_2_ demonstrated a robust combination of high activity and stability.

To rationalize these performance trends, kinetic and thermodynamic analyses were performed. Ni SAC@TiO_2_ exhibited a lower Tafel slope (180 mV dec^−1^) than TiO_2_ (260 mV dec^−1^), reflecting faster charge‐transfer kinetics (Figure 3c). Activation energies (Figure 3f) derived from temperature‐dependent electrochemical impedance spectroscopy (Figure S29a,b) further confirmed the advantage: Ni SAC@TiO_2_ displayed an apparent activation energy of only 16.84 kJ mol^−1^, nearly half of that measured for TiO_2_ (31.09 kJ mol^−1^). This substantial reduction in barrier underscores the enhanced ability of Ni SAC@TiO_2_ to adsorb and activate NH_3_ molecules at the catalyst‐electrolyte interface [21]. Taken together, these results establish Ni SAC@TiO_2_ as a robust and highly active AOR catalyst, where single‐atom Ni incorporation fine‐tunes the Ti─O electronic environment and stabilizes oxygen vacancies. This synergy lowers activation barriers, accelerates charge transfer, and enhances surface stability against poisoning, providing a decisive advantage over nanoparticle‐based and bare supports [53].

### 2.4 Insight into Surface Structural Evolution due to Interaction with NH_3_: In Situ SERS

In situ surface‐enhanced Raman scattering (SERS) was employed to investigate the surface structural evolution [54] of TiO_2_ and Ni SAC@TiO_2_ during the AOR under applied potentials ranging from 1.2 to 2.0 V (vs. RHE), as shown in Figure 2e,f and Figure S30. Both samples exhibited characteristic Raman bands of TiO_2_ at approximately 418–420, 432–433, 450–452, 578–579, and 753 cm^−1^, the latter corresponding to surface‐adsorbed hydroxyl species (*OH). Notably, Ni SAC@ TiO_2_ displayed enhanced peak intensities compared to bare TiO_2_, suggesting more interaction with NH_3_ during AOR [55]. In addition, the ^*^OH peak at 753 cm^−1^ intensified with increasing potential [56], indicating progressive hydroxyl accumulation or surface hydroxylation under oxidative conditions. This effect was more pronounced for Ni SAC@TiO_2_, highlighting the role of Ni single atoms, which enhanced Ti‐O covalency, promoting ^*^OH, which helps in more dehydrogenation than TiO_2_. These results demonstrate that Ni single‐atom incorporation facilitates the generation of OH species and modifies the surface chemistry of TiO_2_ under electrochemical conditions.

### 2.5 Insight Into Mechanism and Deactivation: In Situ ATR‐SEIRAS and XPS of Used Catalysts

**FIGURE 4:**
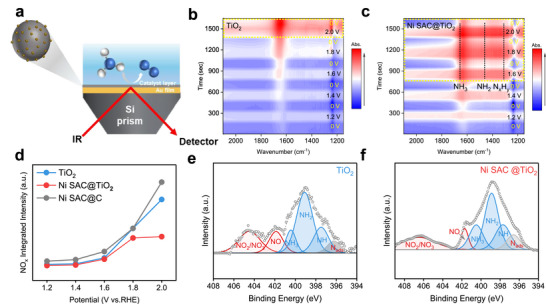
In situ ATR‐SEIRAS analysis of AOR. (a) Schematic illustration of the ATR‐SEIRAS setup using a Si prism coated with an Au film. In situ ATR‐SEIRAS spectra during multiple‐step chronoamperometry of AOR on (b) TiO_2_ and (c) Ni SAC@ TiO_2_. (b) Ni SAC@ TiO_2_ and (c) TiO_2_. (d) Integrated SEIRA peak intensities of NO (1540–1570 cm^−1^), NO_2_ (1230–1250 cm^−1^), and NO_3_^−^ (1380–1390 cm^−^^1^) as a function of applied potential and for Ni SAC@TiO_2_ at 1.6 V vs RHE (e). (f–h) Ex situ XPS analysis after applying 1.6 V vs RHE for 30 min on (e) TiO_2_, and (f) Ni SAC@ TiO_2_.

To elucidate the catalytic mechanism and deactivation underlying ammonia oxidation, in situ attenuated total reflection‐surface enhanced infrared absorption spectroscopy (ATR‐SEIRAS) was employed to monitor surface intermediates on TiO_2_, Ni SAC@TiO_2_, and Ni SAC@C (Figure 4). In situ ATR‐SEIRAS measurements were conducted during linear sweep voltammetry (LSV) on TiO_2_, Ni SAC@TiO_2_, and Ni SAC@C electrodes. As shown in the spectra (Figure S32), an increase in the N*_x_* H*_y_*‐related band at ∼1260 cm^−1^ was observed above 0.3 V, indicating the initial step of dehydrogenation for ammonia oxidation reaction. Subsequently, the emergence of the NH_2_ band at ∼1430 cm^−1^ above 0.9 V suggests that the oxidation proceeds via NH dimerization, following the Gerischer‐Maurer mechanism. At potentials above 1.5 V, a prominent NO peak appears at ∼1530 cm^−1^. Notably, Ni SAC@C exhibits a pronounced increase in NO intensity, indicating the accumulation of this poisoning intermediate. With further increase in potential (above 1.8 V), a distinct NO_2_ peak (∼1240 cm^−1^) and a NO_3_ peak at ∼1380 cm^−1^ become evident, reflecting progressive oxidation of nitrogen species [57, 58, 59, 61]. Among these catalysts, Ni SAC@TiO_2_ shows the least accumulation of NO*_x_*‐related bands compared to TiO_2_ and Ni SAC@C. This suggests that Ni SAC@TiO_2_ effectively suppresses the formation of strongly adsorbed NO*_x_* species while promoting selective NH_3_ oxidation through NH*_x_*‐NH*_y_* coupling intermediates. Alongside the conversion of NH_3_ to N_2_, the generation of NO*_x_* intermediates inherently occurs during the ammonia oxidation reaction. To gain deeper insight into surface reactions during the AOR, in situ ATR‐SEIRAS spectra were further recorded under multiple‐step chronoamperometry (CA), in which the potential was alternately held at 0.05 V and stepped up to 1.2, 1.4, 1.6, 1.8, and 2.0 V. During this potential cycling, Ni SAC@TiO_2_ exhibited distinct vibrational features corresponding to NH_3_‐derived species, including N*_x_* H*_y_* (∼1260 cm^−1^) and NH_2_ (∼1430 cm^−1^). In contrast, TiO_2_ showed negligible spectral response under identical conditions (Figure 4b,c), highlighting the essential role of Ni SAC in active sites modulation in facilitating AOR. As demonstrated previously, the formation of NO*_x_* species is an unavoidable aspect of the ammonia oxidation pathway. Despite this, spectra collected during CA revealed that Ni SAC@TiO_2_, while exhibiting the highest catalytic activity among the tested samples, generated significantly less NO*_x_*‐related signals compared to both TiO_2_ and Ni SAC@C (Figure 4d; Figure S34). In particular, when 1.6 V was applied, Ni SAC@TiO_2_ produced lower amounts of NO species, as shown in the quantitative analysis in Figure 4d. This suggests that the catalyst not only suppresses the formation of NO*_x_*, a well‐known poisoning intermediate, but also promotes the efficient conversion of ammonia via NH*_x_* intermediates into N_2_. Suppressing NO*_x_* accumulation is crucial for maintaining catalytic stability and long‐term selectivity. This trend is further supported by ex situ N 1s XPS analysis following CA at 1.6 V for 30 min (Figure 4e,f; Figure S35). While TiO_2_ and Ni SAC@C displayed pronounced signals corresponding to NO and NO_2_, the Ni SAC@TiO_2_ surface showed dominant peaks associated with NH_2_ and other NH_3_‐derived species, along with significantly reduced NO*_x_* signals [60, 62, 63]. These results indicate that Ni SAC@TiO_2_ not only suppresses the accumulation of poisoning species but also promotes the dehydrogenation of NH_3_ to form N_2_. Overall, the combined ATR‐SEIRAS and XPS analyses identify Ni SAC@TiO_2_ as an efficient AOR catalyst capable of suppressing toxic NO*_x_* intermediate formation while enhancing NH_3_‐to‐NH*_x_* transformation. This behavior is particularly advantageous for electrochemical systems designed to use ammonia as a hydrogen carrier, allowing for clean, selective, and efficient hydrogen extraction with minimal generation of undesirable byproducts.

### 2.6 Theoretical Insight

To rationalize the experimentally observed activity and selectivity trends, reaction free‐energy profiles for both N_2_ and NO_2_ formation were systematically evaluated on three representative surfaces: pristine TiO_2_, Ni SAC@TiO_2_, and Ni SAC@C. Details of the model construction are provided in the Section 4.9 and Figures S36–S39. For N_2_ formation, two mechanistic routes were considered: (i) the Gerischer–Mauerer (G–M) mechanism, which proceeds via NH*_x_*‐NH*_y_* surface coupling between partially dehydrogenated species (0 ≤ *x*, *y* ≤ 2), and (ii) the Oswin–Salomon (O–S) mechanism, which involves N‐N homocoupling following extensive dehydrogenation. Multiple coupling intermediates (^*^NH_2_‐NH_2_, ^*^NH‐NH_2_, ^*^NH‐NH, ^*^N‐NH, and ^*^N‐N) were examined to identify the most favorable reaction coordinates on each surface.

**FIGURE 5:**
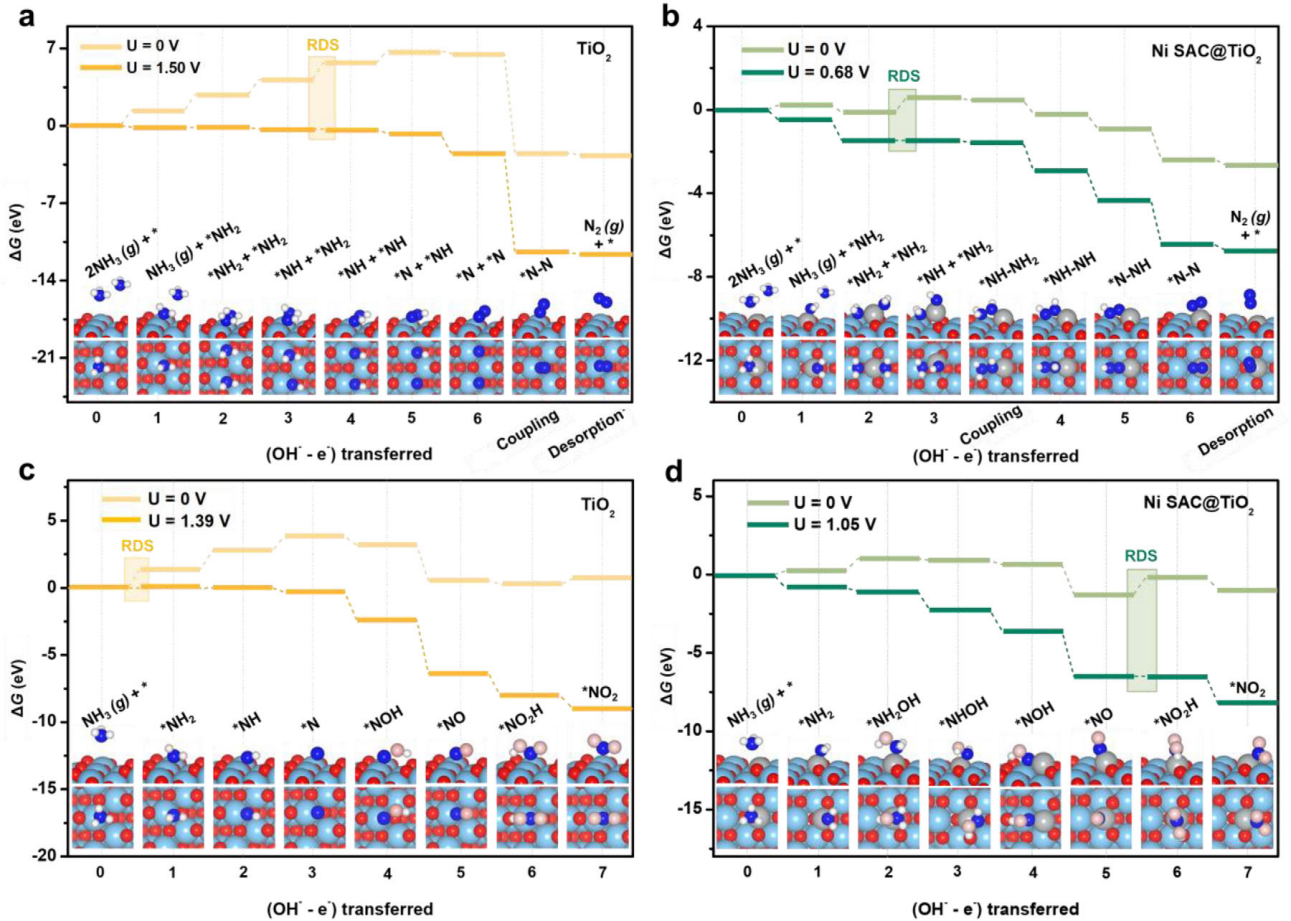
Free‐energy profiles and optimized geometries of key intermediates along the minimum‐energy pathways of the AOR at pH 11. (a) Pristine TiO_2_ following the O–S mechanism toward N_2_ formation. (b) Ni SAC@TiO_2_ following the G–M mechanism toward N_2_ formation. (c) Pristine TiO_2_ following the pathway toward NO_2_ formation. (d) Ni SAC@TiO_2_ following the pathway toward NO_2_ formation. Light blue, red, gray, blue, light pink, and white spheres denote Ti, lattice O, Ni, N, adsorbate O, and H atoms, respectively.

On the pristine TiO_2_ surface, the G–M pathway is energetically unfavorable due to the limited spatial separation between Ti active sites, which prevents effective on‐surface coupling of NH*_x_*‐NH*_y_* intermediates. As a result, N_2_ formation proceeds exclusively via the O–S route. In this pathway, the ^*^NH + ^*^NH_2_ → ^*^NH + ^*^NH step constitutes the rate‐determining step (RDS) and exhibits a high free‐energy barrier of 1.50 eV (Figure 5a). This large barrier is consistent with the experimentally observed low intrinsic AOR activity and poor N_2_ selectivity of pristine TiO_2_. Incorporation of isolated Ni atoms onto the TiO_2_ surface modifies the reaction landscape by creating a Ni─O─Ti dual‐site motif with complementary electronic characteristics. On the Ni SAC@TiO_2_ surface, the O–S pathway remains accessible but with a reduced RDS barrier of 1.26 eV (Figure S40). More importantly, the G–M pathway becomes energetically preferred (Figure 5b). In this case, the dehydrogenation step (^*^NH_2_ + ^*^NH_2_ → ^*^NH + ^*^NH_2_) serves as the RDS with a significantly lower barrier of 0.68 eV. The interfacial Ni─O─Ti configuration stabilizes NH*_x_* ─NH*_y_* coupling intermediates and lowers the energetic penalty for their formation, which is consistent with the NH*_x_*‐related coupling features observed by in situ ATR‐SEIRAS during AOR. This dual‐site effect provides a clear mechanistic basis for the enhanced activity of Ni SAC@TiO_2_. On the Ni SAC@C surface, the G–M pathway offers an energetically viable route for NH_3_ activation, with a low dehydrogenation barrier of 0.66 eV (Figure S41). However, N_2_ desorption is energetically unfavorable, with a desorption free energy of 0.83 eV, which is expected to hinder product release and limit catalytic turnover. In addition, the O–S mechanism remains kinetically limited by the dehydrogenation step (^*^N + NH → ^*^N + ^*^N), exhibiting a high energy barrier of 1.32 eV (Figure S42).

To evaluate product selectivity, reaction pathways leading to nitrite (NO_2_) formation were also investigated. Hydroxylation of key NH*_x_* intermediates (^*^NH_2_, ^*^NH, and ^*^N) to form ^*^NH_2_OH, ^*^NHOH, and ^*^NOH species was considered. The most favorable reaction coordinates for each surface are summarized in Figure 5c,d and Figure S43. On the pristine TiO_2_, NO_2_ formation requires complete dehydrogenation to ^*^N, resulting in a high RDS barrier of 1.39 eV (Figure 5c). On the Ni SAC@TiO_2_, incorporation of Ni shifts the RDS to the oxidation of ^*^NO to ^*^NO_2_H and lowers the barrier to 1.05 eV (Figure 5d). In contrast, the Ni SAC@C exhibits the lowest barrier for NO_2_ formation (0.78 eV), rationalizing its stronger tendency toward NO*_x_* accumulation observed experimentally (Figure S34).

Overall, although both Ni SAC@TiO_2_ and Ni SAC@C enhance AOR relative to pristine TiO_2_, they exhibit distinct catalytic behaviors. Ni SAC@TiO_2_ achieves a favorable balance of activity and selectivity by preferentially promoting N_2_ formation through the G–M pathway (RDS = 0.68 eV), whereas pristine TiO_2_ is kinetically limited and Ni SAC@C is more susceptible to product inhibition and deactivation. These mechanistic insights are consistent with our experimental observations.

To further elucidate the origin of the Ni─TiO_2_ synergy, the projected density of states (PDOS) and charge‐density difference analyses were performed (Figure S44). The Ni atom in Ni SAC@TiO_2_ adopts a square‐planar coordination with four surface O atoms, forming robust Ni─O bonds. The charge‐density difference map shows electron depletion around Ni and accumulation along the Ni─O bonding region, indicating charge transfer from Ni to the surrounding O atoms. Strong hybridization between Ni 3*d* and O 2*p* states indicates the covalent nature of the Ni─O interaction, which contributes to the stabilization of isolated Ni atoms against migration or aggregation. In addition, Ni incorporation introduces Ni‐derived states near the Fermi level, narrowing the band gap and enhancing electronic conductivity. This electronic modulation facilitates charge transfer and supports the enhanced AOR kinetics observed for Ni SAC@TiO_2_.

## 3 Conclusion

In summary, we have demonstrated that Ni single‐atom incorporation into TiO_2_ enables covalent modulation of the Ti‐O framework, establishing a powerful strategy to unlock efficient and durable catalysts for the electrochemical ammonia oxidation reaction. The Ni SAC@TiO_2_ catalyst nearly doubled the activity of pristine TiO_2_ and exhibited outstanding stability, retaining over 98% of its performance after 2000 accelerated cycles. Advanced spectroscopic analyses, including hard and soft XAS and XPS studies, revealed strong metal‐support interactions that enhance Ti‐O covalency and create highly active sites, while in situ SERS confirmed that this structural tuning promotes strong reactant and intermediates interaction, and in situ ATR‐SEIRAS studies affirmed NH*_x_*‐NH*_y_* mediated pathways and suppressed the accumulation of deactivating NO*_x_* species. Complementary DFT calculations provided atomic‐level insights into the origin of enhanced reactivity, selectivity, and durability. This work establishes single‐atom modulation of covalency as a broadly applicable approach for re‐engineering TiO_2_‐based catalysts, bridging the gap between fundamental design principles and enhanced catalytic performance. Beyond advancing AOR catalysis, the strategy outlined here offers a generalizable framework for tailoring electronic structures and reaction pathways in transition metal oxides, paving the way toward scalable, non‐noble metal catalysts for energy and environmental applications.

## 4 Experimental Section

### 4.1 Materials

All chemicals were used as received without further purification. Titanium (IV) n‐butoxide (TBOT), tetraethyl orthosilicate (TEOS), nickel (II) chloride hexahydrate (NiCl_2_·6H_2_O), carbon powder, and polyvinylpyrrolidone (PVP, Mw ≈ 55 000) were obtained from Sigma–Aldrich. Acetonitrile, ethanol, sodium hydroxide (NaOH), and aqueous ammonia solution (28–30 wt.%) were purchased from Samchun Chemical.

### 4.2 Catalysts Preparation

#### 4.2.1 Synthesis of TiO_2_ and Ni SAC@TiO_2_

TiO_2_ and Ni SAC@TiO_2_ were synthesized via a thermodynamic redistribution approach using SiO_2_ particles as sacrificial templates. SiO_2_ particles were first prepared by a sol–gel method: 0.86 mL of TEOS was added to a mixed solution of H_2_O (4.3 mL), ethanol (23 mL), and aqueous ammonia (0.6 mL), followed by stirring for 12 h. The resulting SiO_2_ particles were collected by centrifugation, washed with ethanol and water, and dispersed in 40 mL of aqueous ethanol via 10 min sonication. Subsequently, acetonitrile (14 mL) and ammonia (0.4 mL) were added. Separately, a precursor solution containing TBOT (0.8 mL), aqueous ethanol (6 mL), and acetonitrile (2 mL) was prepared and added dropwise to the SiO_2_ suspension. After 12 h of stirring, the SiO_2_@TiO_2_ product was isolated, washed, and dispersed in 40 mL of water. A stoichiometric amount of NiCl_2_·6H_2_O was then introduced, followed by 3 h of stirring. After washing, 0.4 g of PVP was added and stirred for another 12 h to ensure surface adsorption. The PVP‐coated particles were centrifuged and dispersed in 46 mL of ethanol and 8.6 mL of water with 10 min of sonication. A second SiO_2_ shell was formed by adding aqueous ammonia (1.2 mL) and TEOS (1.6 mL), followed by a 4 h reaction. The resulting product was washed, dried at 80°C overnight, and calcined in air at 950°C for 3 h (ramp rate: 2°C/min). After cooling, the sample was etched in 0.5 m NaOH at 90°C for 8 h to remove the SiO_2_, yielding Ni SAC@TiO_2_. TiO_2_ was synthesized using the same procedure but omitting the nickel precursor step.

#### 4.2.2 Ni NP@TiO_2_ Synthesis

Ni nanoparticle‐loaded TiO_2_ (Ni NP@TiO_2_) was synthesized via a chemical reduction method. Briefly, 2 mL of 0.1 m NiCl_2_·6H_2_O solution was added to a 100 mL round‐bottom flask, followed by the addition of 100 mg of pre‐synthesized TiO_2_ under vigorous magnetic stirring (800 rpm) for 10 min to facilitate the adsorption of Ni^2^^+^ ions onto the TiO_2_ surface. Subsequently, 45 mg of NaBH_4_ was added as a reducing agent. The appearance of gas bubbles indicated the reduction process. Stirring was continued for ∼8 min until gas evolution ceased. The resulting black suspension was collected and dried at 80°C overnight to yield the Ni NP@TiO_2_ catalyst.

#### 4.2.3 Ni SAC@C Synthesis

Ni single‐atom catalyst supported on carbon (Ni SAC@C) was synthesized via a freeze‐drying and thermal annealing route. A dispersion of commercially available carbon powder (4 mg mL^−^^1^, 100 mL total volume) was prepared, into which a calculated amount of NiCl_2_·6H_2_O was added. The mixture was stirred for 24 h to ensure uniform adsorption of Ni^2^^+^ ions. The resulting suspension was freeze‐dried for 24 h, yielding a brownish solid, which was subsequently annealed at 750°C for 3 h under an Ar atmosphere to form Ni SAC@C.

### 4.3 Characterization

X‐ray absorption fine structure (XAFS) measurements were conducted at the 8C NanoProbe XAFS beamline (BL8C) of the Pohang Light Source (PLS‐II). Ti K‐edge and Ni K‐edge spectra were acquired in both transmission and fluorescence modes. The acquired XAFS data were processed using the ATHENA module within the IFEFFIT software suite. Fourier‐transformed extended X‐ray absorption fine structure (FT‐EXAFS) fitting was carried out using Artemis. High‐angle annular dark‐field scanning transmission electron microscopy (HAADF‐STEM) imaging, STEM‐energy dispersive X‐ray spectroscopy (STEM‐EDS) elemental mapping, and STEM ‐ electron energy loss spectroscopy (STEM ‐ EELS) core loss data were performed at 300 kV using a double aberration‐corrected STEM (Spectra Ultra, Thermo Fisher Scientific, NFEC‐2023‐01‐284572) equipped with Ultra‐X EDS detectors, housed at the KENTECH Center for Shared Research Facilities. Data were processed with Velox software (Thermo Fisher Scientific) and Digital Micrograph (Gatan). High‐resolution field‐emission transmission electron microscopy (HR‐TEM) with Cs correction was used for imaging, alongside selected area electron diffraction (SAED) for interplanar spacing analysis. Powder X‐ray diffraction (PXRD) patterns were collected using Cu Kα radiation (RIGAKU D/MAX 2500 diffractometer). X‐ray photoelectron spectroscopy (XPS, Axis‐Nova, Kratos) was employed to determine the chemical states of elements. Soft X‐ray absorption spectroscopy (XAS) was carried out at the 10A2 high‐resolution photoemission spectroscopy (HR‐PES II) beamline of PLS‐II.

### 4.4 In Situ ATR‐SEIRAS Analysis

For ATR‐SEIRAS experiments, catalyst inks were made using 1 mg of TiO_2_, Ni SAC @ TiO_2_, Ni SAC @C, 600 µL of isopropanol (IPA), and 6 µL of Nafion solution, followed by extensive sonication. Five hundred microliters of the resultant ink were spray‐coated onto a hemicylindrical silicon prism (Pike Technologies) that had been pre‐coated with electroless deposited gold, functioning as the working electrode. The ATR‐SEIRAS measurements were performed in a three‐electrode setup utilizing an Ag/AgCl reference electrode (BASi, 3 m NaCl) and a platinum wire counter electrode. A Fourier‐transform infrared spectrometer (VERTEX 80v, Bruker) with a variable‐angle specular reflectance accessory (Veemax III, Pike Technologies) and a liquid‐nitrogen‐cooled mercury cadmium telluride (MCT) detector was utilized to acquire spectra. All spectra were obtained in absorbance mode with a resolution of 4 cm^−^^1^. Absorbance is defined as:

$$A=-\log\left(I/I_{0}\right)$$

where I and I_0_ represent the spectral intensities at an applied potential and open‐circuit potential (OCV), respectively. (Figure S31) Linear sweep voltammetry (LSV) was performed from 0 V to 2.0 V at a scan rate of 20 mV s^−^^1^. A multiple‐step chronoamperometry (CA) sequence was employed to examine potential‐dependent interfacial behavior by alternating the potentials of 0.05, 1.2, 1.4, 1.6, 1.8, and 2.0 V, each maintained for 150 s (Figure S33). All measurements were conducted repeatedly to ensure reliability.

### 4.5 In Situ SERS Analysis

In situ SERS measurements were conducted using a Renishaw inVia Qontor system equipped with a 532 nm Nd: YAG laser, a 50× objective lens (NA = 0.55), an 1800 lines/mm grating monochromator, and a CCD detector. Each Raman spectrum was acquired with an integration time of approximately 30 s. A Spectro‐electrochemical cell with a quartz window was employed for in situ SERS measurements. A Pt wire and an Ag/AgCl (saturated KCl) electrode were used as the counter and reference electrodes, respectively. TiO_2_ and Ni SAC@TiO_2_ films coated on Au‐coated silicon wafers served as the working electrodes. During measurements, the Ar‐saturated electrolyte (0.1 m NaClO_4_ + 0.1 m NH_3_) was circulated through the cathodic compartment at a flow rate of 0.5 mL min^−^^1^ using a peristaltic pump. Potential‐dependent Raman spectra were collected via multi‐step chronoamperometry, applying sequential potentials from 1.2 to 2.0 V vs. RHE in 0.2 V increments, each held for 1 min.

### 4.6 Electrochemical AOR Measurements

Electrochemical measurements were performed using a Metrohm Autolab potentiostat in a custom‐built H‐type three‐electrode glass cell, comprising a 25 mL volumetric chamber, separated by a Nafion 117 membrane. A platinum foil and an Ag/AgCl electrode (BASi, 3 m KCl) served as the counter and reference electrodes, respectively. Ammonia oxidation was carried out in Ar‐saturated 0.1 m NaClO_4_ electrolyte (pH ∼ 11.2) containing varying concentrations of aqueous NH_3_ (0.1, 0.2, and 0.3 m). The working electrode was prepared by sonicating a dispersion of 1 mg catalyst and 10 µL Nafion in 1 mL of water/isopropanol (4:1, v/v) for 60 min. Then, 50 µL of the homogeneous ink was drop‐cast onto a glassy carbon electrode (GCE) to achieve a catalyst loading of 0.1 mg cm^−^^2^. Linear sweep voltammetry (LSV) and Cyclic Voltammetry (CV) were conducted from 0 to 2 V vs. RHE at a scan rate of 10 mV s^−^^1^. Long‐term stability tests were performed in 0.1 m NaClO_4_ with 0.1 m NH_3_ by cycling the potential between 0 and 2 V vs. RHE at 50 mV s^−^^1^ for a predetermined number of cycles.

### 4.7 Faradaic Efficiency Caculations

The Faradaic efficiencies (FE) of the products were calculated according to:

$$\%\;FE=\left(eF*\frac{n}{Q}\right)*100$$

*FE* = Faradaic efficiency (%), *e* = moles of electrons, *Q* = total obtained charge (C), *n* = moles of products, *F* = Faraday's constant = 96 485 C mol^−1^.

The Faradaic efficiency (FE) of each product was calculated based on the total charge passed during electrolysis and the amount of product formed. The number of electrons transferred for each product was determined according to the corresponding reaction stoichiometry. *FE* was calculated using the equation above, where *n* is the amount of product, *e* is the number of electrons involved, *Q* is the total charge, and *F* is Faraday's constant (96485 C mol^−1^).

### 4.8 Determination and Quantitation of NO_3_^−^ by UV–vis Spectrophotometry

The concentration of nitrate (NO_3_^−^) was determined using UV–vis spectrophotometry. Briefly, 1.0 mL of the electrolyte was transferred to a 20 mL volumetric flask and diluted to volume with deionized water. Subsequently, 1.0 mL of the diluted solution was mixed with 20 µL of 1 m HCl. After standing for 10 min to ensure complete protonation and stabilization, the UV–vis absorption spectrum was recorded. Nitrate concentration was quantified using the difference in absorbance at 220 and 275 nm (A_220_−A_275_). A calibration curve was constructed using potassium nitrate (KNO_3_) standard solutions of known concentrations following a previously reported method [64, 65].

### 4.9 Computational Details

All spin‐polarized DFT calculations were performed using the Vienna *Ab* initio Simulation Package (VASP, version 5.4.4) [66], employing the Perdew–Burke–Ernzerhof (PBE) functional within the generalized gradient approximation (GGA) framework [67]. Long‐range van der Waals interactions were accounted for using the DFT‐D3 dispersion correction scheme with zero damping [68]. Core–valence electron interactions were described by the projector‐augmented wave (PAW) method [69], and a plane‐wave basis set with a kinetic energy cutoff of 520 eV was employed. Geometry optimizations were carried out until the total energy change between successive ionic steps was below 10^−6^ eV, and residual forces on each atom were less than 0.01 eV/Å. Electronic states were treated using the Methfessel‐Paxton smearing method with a smearing width of 0.03 eV. A vacuum region exceeding 20 Å was introduced along the surface‐normal direction to eliminate spurious interactions between periodic images, and dipole corrections were applied. For electronic structure analysis, the standard HSE06 hybrid functional was additionally employed [70].

The anatase TiO_2_ unit cell was optimized to reproduce experimental lattice parameters (Figure S36) [71]. Based on this optimized structure, a (1 × 2) TiO_2_ (101) surface slab consisting of three atomic layers was constructed (Figure S37). During structural relaxation, the bottom two layers were fixed at their bulk positions, while the top layer and all adsorbed species were fully relaxed. A 3 × 4 × 1 Monkhorst‐Pack *k*‐point mesh was employed. The Ni SAC@TiO_2_ was modeled by placing a single Ni atom at several distinct surface sites on the TiO_2_ (101) slab, including (i) an O*_2c_* site, where the Ni atom is adsorbed directly atop a two‐coordinated bridging O atom, (ii) a bridge‐O*_2c_* site, where the Ni atom is located at a bridging position interacting with adjacent surface O atoms, and (iii) a square‐planar site, in which the Ni atom is incorporated into the surface lattice and coordinated by four surrounding O atoms (Figure S38). Among these configurations, the square‐planar site was found to be the most energetically favorable and was therefore adopted for subsequent calculations. For the Ni SAC@C model, 4 × 4 graphene supercell containing 32 carbon atoms was constructed, and a single Ni atom was placed at three representative adsorption sites: (i) the hollow site, where the Ni atom is centered in a hexagonal C ring, (ii) the bridge site, where the Ni atom is located above a C─C bond, and (iii) the top site, where the Ni atom is positioned directly atop a carbon atom (Figure S39). A 4 × 4 × 1 Monkhorst‐Pack *k*‐point grid was used. Among these configurations, the hollow site was identified as the most stable and was therefore adopted for all subsequent analyses.

The Gibbs free energy (*G*) of each states was calculated according to:

G=E+ZPE−TΔS

where *E* is the DFT total energy, *ZPE* is the zero‐point energy correction, and *T*∆*S* is the entropic contribution. All thermochemical corrections for both gas‐phase and adsorbed species are summarized in Tables S6–S9. Entropic contributions for gas‐phase molecules were taken from the National Institute of Standards and Technology (NIST) Chemistry WebBook [72]. Electrochemical free‐energy changes were evaluated using a previously reported approach [73].

## Conflicts of Interest

The authors declare no conflicts of interest.

## Supporting information




**Supporting File**: advs73775‐sup‐0001‐SuppMat.docx.

## Data Availability

The data that support the findings of this study are available from the corresponding author upon reasonable request.
