# Supplementary material for: Ni Single‐Atom Modulation of Ti‐O Covalency Boosts Ammonia Oxidation Electrocatalysis
Source: Adv Sci (Weinh). 2026 Jan 20;13(17):e21932. doi: 10.1002/advs.202521932 (PMC13042473; doi:10.1002/advs.202521932)
Supplement: Supplementary file 1 — Supporting File: advs73775‐sup‐0001‐SuppMat.docx. [file ADVS-13-e21932-s001.docx]

**Supporting Information**

***for***

**Ni Single-Atom Modulation of Ti-O Covalency Boosts Ammonia Oxidation Electrocatalysis**

*Subhash Chandra Shit,* *Dayoung Kwon, Nhi Thi Yen Phan,* *Hyo Won Kim, Jucheol Park, Jeong-hyeon Lee, Hyeyoung Shin^*^, Wooyul Kim^*^*

S. C. Shit, D. Kwon, H. W. Kim, W. Kim

Department of Energy Engineering

Korea Institute of Energy Technology (KENTECH)

Naju 58330, Republic of Korea.

E-mail: [wkim@kentech.ac.kr](mailto:wkim@kentech.ac.kr)

J. Park, J. Lee

Center for Shared Research Facilities

Korea Institute of Energy Technology (KENTECH)

Naju 58330, Republic of Korea.

*N. T. Y. Phan, H. Shin*

Graduate School of Energy Science and Technology (GEST)

Chungnam National University

Daejeon 34134, Republic of Korea

E-mail: [shinhy@cnu.ac.kr](mailto:shinhy@cnu.ac.kr)

S. C. Shit, D. Kwon and N. T. Y. Phan contributed equally to this work

|  | Signal (e-) | Comp. (at. %) | Comp. (wt. %) | Area Dens.  (at./nm^2^) | Vol. Dens.  (at./nm^3^) | X-section  (barns) | X-section  Model |
| --- | --- | --- | --- | --- | --- | --- | --- |
| Ti L | 66.8e+03 | 99.1 | 98.9 | 920 | 60 | 9.2e+03 | Hartree-Slater |
| Ni L | 130 | 0.9 | 1.1 | 8 | 0.5 | 2.0e+03 | Hartree-Slater |

|  | Signal (e-) | Comp. (at. %) | Comp. (wt. %) | Area Dens.  (at./nm^2^) | Vol. Dens.  (at./nm^3^) | X-section  (barns) | X-section  Model |
| --- | --- | --- | --- | --- | --- | --- | --- |
| Ti L | 74.6e+03 | 100.0 | 100.0 | 1040 | 74 | 9.4e+03 | Hartree-Slater |
| Ni L | 0 | 0.0 | 0 | 0 |  | 2.1e+03 | Hartree-Slater |

**Figure S1.** Electron energy loss spectroscopy (EELS) characterization of Ni SAC@TiO_2_. Images and spectroscopic data represent areas (a) with Ni and (b) without Ni atoms.

**Figure S2.** (a) HAADF STEM image and (b, c) Electron energy loss spectroscopy (EELS) characterization of Cu SAC@TiO_2_. Images and spectroscopic data represent areas with Cu atoms.

**Figure S3.** (a) HAADF STEM image and (b, c) Electron energy loss spectroscopy (EELS) characterization of Co SAC@TiO_2_. Images and spectroscopic data represent areas with Co atoms.

**Figure S4.** STEM-EDS elemental mapping of TiO_2_ showing the homogeneous distribution of Ti and O elements.

**Figure S5.** STEM-EDS elemental mapping of Cu SAC@TiO_2_ showing the homogeneous distribution of Cu, Ti and O elements.

**Figure S6.** STEM-EDS elemental mapping of Co SAC@TiO_2_ showing the homogeneous distribution of Co, Ti and O elements.

**Figure S7.** STEM-EDS elemental mapping of Ni SAC@C showing the uniform distribution of Ni and C elements.

**Figure S8.** STEM-EDS elemental mapping of Ni NP@TiO_2_ showing the homogeneous distribution of Ni, Ti and O elements.

**Figure S9.** (a) Low-resolution TEM image of TiO_2_, and (b) HR-TEM image of TiO_2_, where lattice fringes correspond to the anatase TiO_2_ (103) plane.

**Figure S10.** (a) Low-resolution TEM image of Ni SAC@TiO_2_. (b) High-resolution TEM (HR-TEM) image of Ni SAC@TiO_2_, showing lattice fringes corresponding to the anatase TiO_2_ (101) plane; the inset displays the selected area electron diffraction (SAED) pattern.

**Figure S11.** (a) Low-resolution TEM image of Ni SAC@C, and (b) HR-TEM image of Ni SAC@C, where lattice fringes correspond to the carbon (002) plane.

**Figure S12.** (a) Low-resolution TEM image of Ni NP@TiO_2_. (b) High-resolution TEM (HR-TEM) image of Ni NP@TiO_2_, (b) showing lattice fringes corresponding to the rutile TiO_2_ (101) plane; (c, d) highlighting amorphous Ni particle

**Figure S13.** Powder X-ray diffraction (PXRD) patterns of TiO_2_-based catalysts. (a) PXRD patterns of pristine TiO_2_, Co SAC@TiO_2_, and Cu SAC@TiO_2_. (b) PXRD patterns of pristine TiO_2_, Ni SAC@TiO_2_, and Ni NP@TiO_2_, showing characteristic peaks corresponding to the anatase and rutile phases of TiO_2_.

**Figure S14.** High-resolution X-ray photoelectron spectroscopy (XPS) analysis. Ti 2p spectra with peak fitting for (a) TiO_2_, (b) Ni SAC@TiO_2_, and (c) Ni NP@TiO_2_, showing the chemical state of Ti in each material.

**Figure S15.** High-resolution X-ray photoelectron spectroscopy (XPS) analysis. Ni 2p spectra with peak fitting for (a) Ni SAC@TiO_2_ and (b) Ni NP@TiO_2_, illustrating the oxidation state and chemical environment of Ni in each catalyst.

**Figure S16.** High-resolution X-ray photoelectron spectroscopy (XPS) analysis. O 1s spectra with peak fitting for (a) Ni SAC@C and (b) Ni NP@TiO_2_, showing the different oxygen species and bonding environments.

**Figure S17.** High-resolution X-ray photoelectron spectroscopy (XPS) analysis. C 1s spectra with peak fitting for Ni SAC@C, showing the different carbon bonding environments.

**Figure S18.** (a) Ti K-edge XANES spectra of TiO_2_ and Ni SAC@TiO_2_, showing a change in the white-line intensity, indicative of electronic structure modulation of Ti induced by Ni incorporation. (b) Ni K-edge XANES spectra of Ni SAC@TiO_2_ and Ni SAC@C, compared with NiO as a reference. (c) First derivative of the Ni K-edge XANES spectra in (b), highlighting subtle differences in edge position and spectral features associated with variations in the oxidation state and local coordination environment of Ni.

**Figure S19.** Extended X-ray absorption fine structure (EXAFS) fitting analysis. Ti K-edge EXAFS spectra with fitting of (a) TiO_2_ and (b) Ni SAC@TiO_2_ in R-space. Ni K-edge EXAFS spectra with fitting of (c) Ni SAC@TiO_2_ and (d) Ni SAC@C in R-space.

**Figure S20.** Extended X-ray absorption fine structure (EXAFS) fitting analysis. Ti K-edge EXAFS spectra with fitting of (a) TiO_2_ and (b) Ni SAC@TiO_2_ in k-space. Ni K-edge EXAFS spectra with fitting of (c) Ni SAC@TiO_2_ and (d) Ni SAC@C in k-space.

**Figure S21.** R-space Ni K-edge EXAFS spectra comparing Ni SAC@C with NiO, highlighting the absence of Ni-Ni coordination in Ni SAC@C and confirming atomic dispersion of Ni species.

**Figure S22.** Soft X-ray absorption spectroscopy (XAS) analysis. (a) Ni L-edge XAS spectra of Ni SAC@TiO_2_ and Ni SAC@C, revealing differences in electronic interaction of Ni SAC and supports. (b) C K-edge XAS spectra of Ni SAC@C, highlighting the carbon functionalization.

**Figure S23.** Soft X-ray absorption spectroscopy (XAS) analysis. (a) O K-edge XAS spectra (524-560 eV) of Ni NP@TiO_2_. (b) Ti L-edge XAS spectra of Ni NP@TiO_2._

**Figure S24.** (a) Linear sweep voltammetry (LSV) and (b) cyclic voltammetry (CV) curves of TiO_2_ and M SAC@TiO_2_ (M = Ni, Co, and Cu) electrocatalysts recorded in 0.1 M NaClO_4_ containing 0.1 M NH_3_ at a scan rate of 10 mV s^−1^. The measurements were performed in the potential range of 0–2 V vs. RHE, illustrating the effect of different single-metal centers on the electrochemical behavior toward the ammonia oxidation reaction (AOR).

.

**Figure S25.** Accelerated stress tests (AST) comparing the ammonia oxidation reaction (AOR) stability of (a) Ni SAC@C and (b) Ni NP@TiO_2_ electrocatalysts in the potential range of 0–2 V vs. RHE at a scan rate of 10 mV s^-1^. Measurements were conducted in 0.1 M NaClO_4_ electrolyte containing 0.1 M NH_3_. The solid lines represent the first cycle, while the dotted lines denote the performance after 2000 cycles. The comparison highlights the distinct stability and durability differences relative to Ni SAC@TiO_2_.

**Figure S26.** UV–vis calibration curve for nitrate (NO_3_^-^) concentration determination, where the absorbance was obtained from the difference between the absorbance values at 220 nm and 275 nm.

**Figure S27.** (a) Quantified amounts of O_2_ and N_2_, (b) corresponding Faradaic efficiencies, and (c) quantified amount of generated NO_3_^-^ at 1.5 V vs RHE for TiO_2_, Ni SAC@TiO_2_, Cu SAC@TiO_2_, and Co SAC@TiO_2_

**Figure S28.** (a) Chronoamperometry (CA) curves of TiO_2_ and Ni SAC@TiO_2_ recorded at 1.74 V vs RHE in 0.1 M NaClO_4_ containing 0.1 M NH_3_ for 1 h. (b) Enlarged view of the initial region in (a), highlighting the transient current response.

**Figure S29.** Temperature-dependent electrochemical impedance spectroscopy (EIS) analysis of (a) TiO_2_ and (b) Ni SAC@TiO_2_ electrocatalysts conducted in 0.1 M NaClO_4_ electrolyte containing 0.1 M NH_3_ at temperatures of 20, 30, 40, and 50 °C.

**Figure S30.** *In situ* surface-enhanced Raman scattering (SERS) spectra of (a) TiO_2_ and (b) Ni SAC@TiO_2_ recorded at potentials from 1.2 to 2.0 V vs RHE in 0.1 M NaClO_4_ containing 0.1 M NH_3_.

**Figure S31.** Linear sweep voltammetry (LSV) curves recorded during in situ ATR-SEIRAS measurements of TiO_2_, Ni SAC@TiO_2_, and Ni SAC@C catalysts from 0.05 to 2.0 V vs RHE at a scan rate of 0.02 V s^−1^.

**Figure S32.** ATR-SEIRAS spectra collected during LSV from 0.05 to 2.0 V vs RHE at a scan rate of 0.02 V s^-1^ in 0.1 M NaClO_4_ (dotted lines) and 0.1 M NaClO_4_ containing 0.1 M NH_3_ (colored lines) on (a) TiO_2_, (b) Ni SAC@TiO_2_, and (c) Ni SAC@C, recorded in the 2100-1150 cm^-1^ region. The reference spectrum was obtained at OCV.

**Figure S33.** Applied potential and current profiles of multiple-step chronoamperometry during in situ ATR-SEIRAS analysis.

**Figure S34.** ATR-SEIRA spectra acquired during multiple-step chronoamperometry from 1.2 V to 2.0 V (vs. RHE) in 0.1 M NaClO_4_ containing 0.1 M NH_3_ on (a) TiO_2_, (b) Ni SAC@TiO_2_, and (c) Ni SAC@C, recorded in the 2100-1150 cm^-1^ range. The reference spectrum was obtained at OCV.

**Figure S35.** *Ex situ* XPS analysis after applying 1.6 V vs RHE for 30 min on Ni SAC.


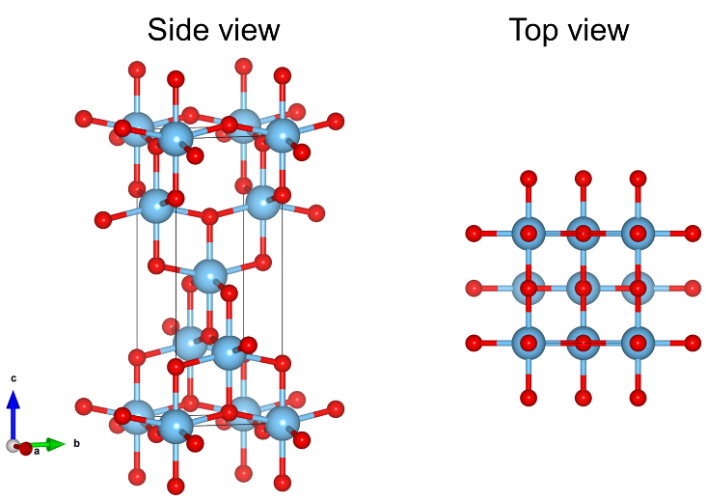


**Figure S36.** Side and top views of bulk anatase TiO_2_ structure. Ti and O atoms are represented by light blue and red spheres, respectively.


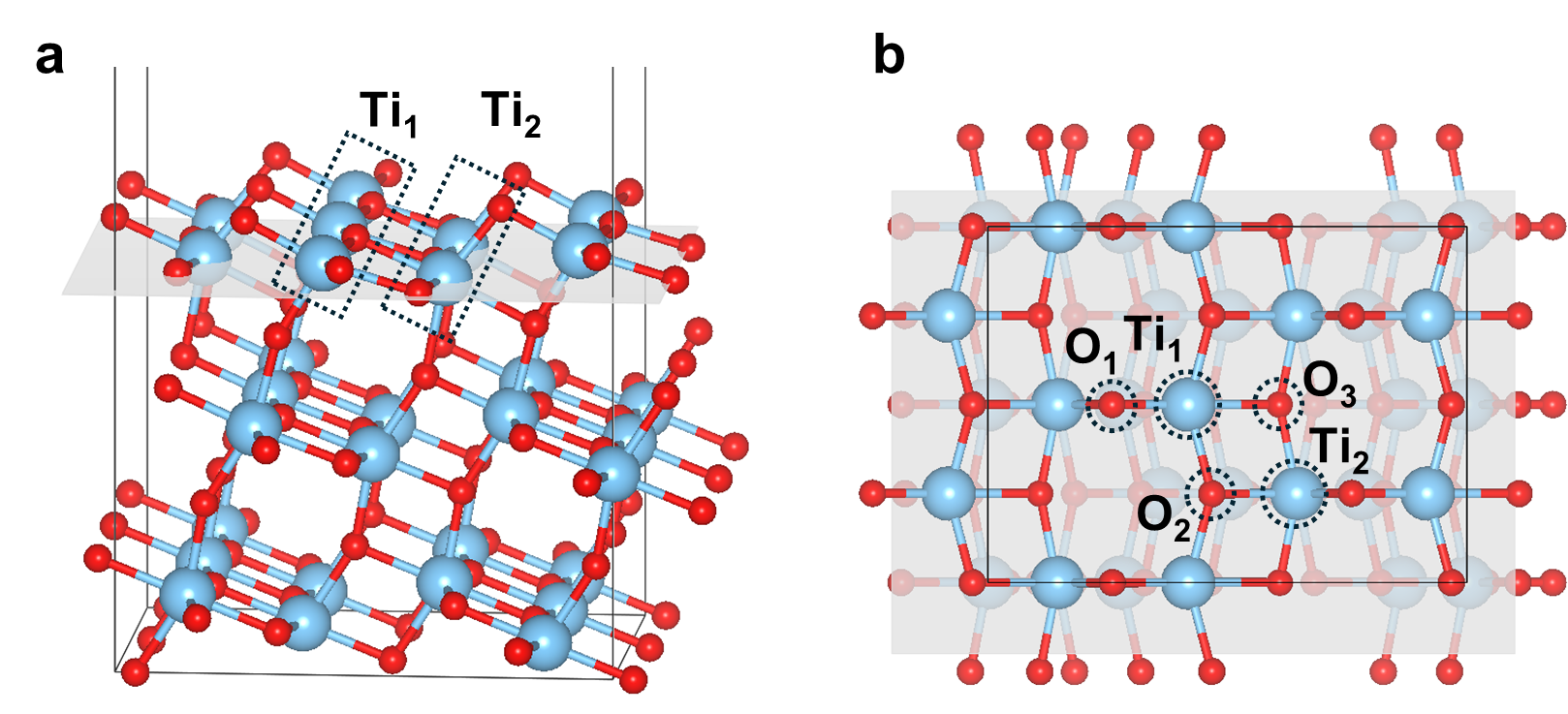


**Figure S37.** Side (a) and top (b) views of TiO_2_ (101) slab model. The surface features penta-coordinated Ti_1_ and octahedrally coordinated Ti_2_ atoms, along with three distinct O sites (O_1_, O_2_, and O_3_). O_1_ corresponds to two-coordinated bridging O atoms, while O_2_ and O_3_ are three-coordinated O sites, which differ in their vertical positions, with O_3_ located higher than O_2_. Ti, and O atoms are represented by light blue and red spheres, respectively.


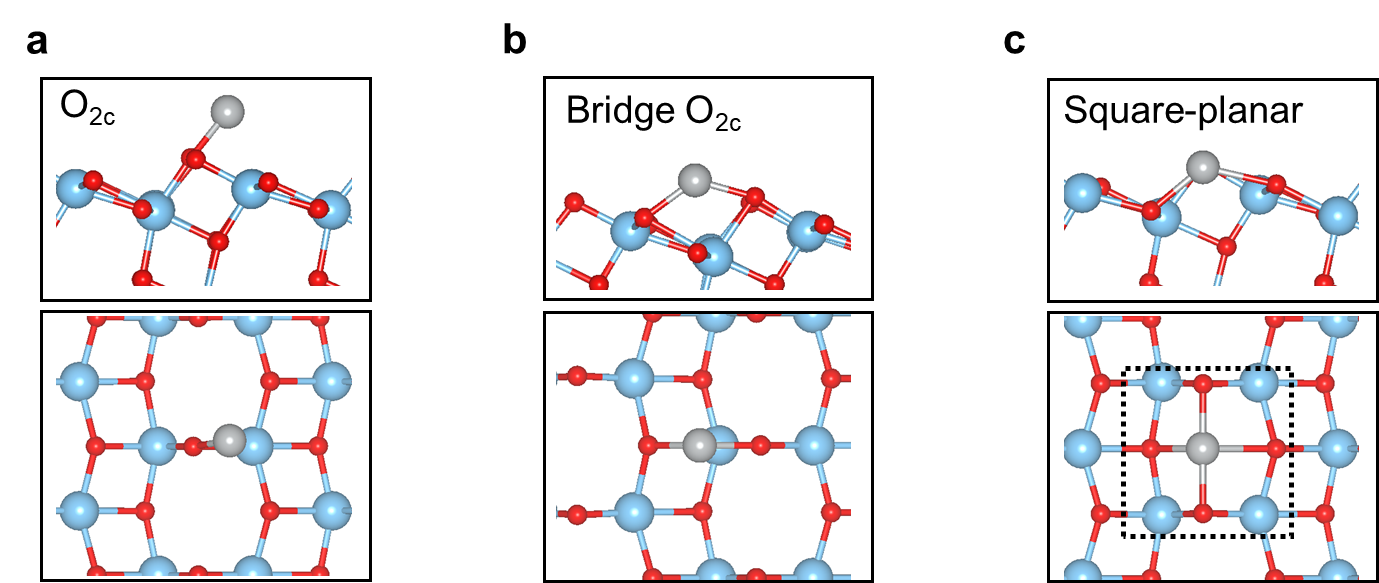


**Figure S38.** Side and top views of the possible geometric configurations of the Ni SAC@TiO_2_. (a) O*_2c_* site: the Ni atom is adsorbed directly atop a two-coordinated bridging O atom (O*_2c_*). (b) Bridge-O*_2c_* site: the Ni atom is located at a bridging position, interacting with adjacent surface O atoms. (c) Square-planar site: the Ni atom is incorporated into the surface lattice, forming a stable square-planar coordination geometry with four surrounding O atoms, as highlighted by the dashed square. Ti, O, and Ni atoms are represented by light blue, red, and gray spheres, respectively.


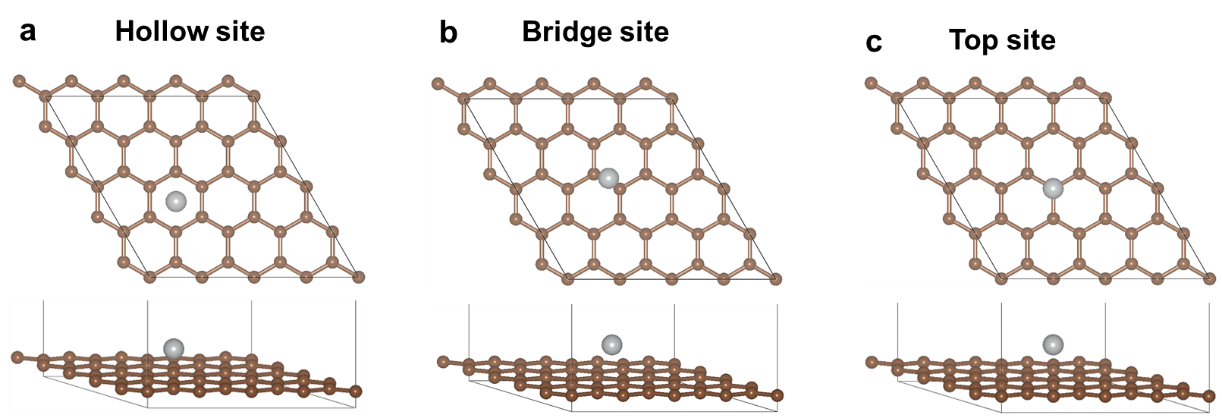


**Figure S39.** Top and side views of possible geometric configurations of the Ni SAC@C. Three binding sites were considered: (a) the hollow site, where the Ni atom is centered in a hexagonal C ring; (b) the bridge site, where the Ni atom is located above a C-C bond; and (c) the top site, where the Ni atom is positioned directly atop a carbon atom. C and Ni atoms are represented by brown and gray spheres, respectively.


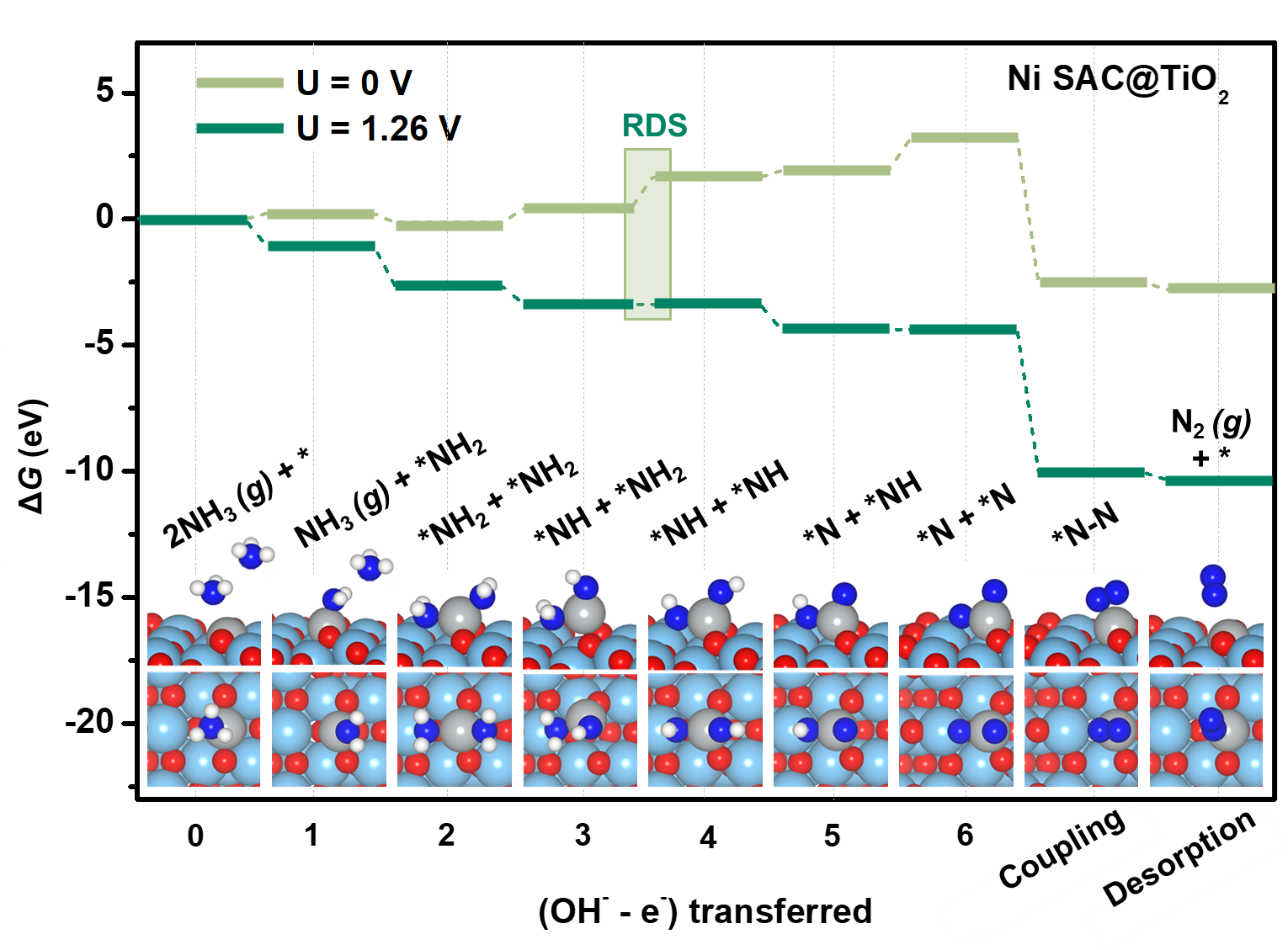
**Figure S40.** Calculated free-energy diagram for the AOR via the O-S pathway toward N_2_ formation on the Ni SAC@TiO_2_ surface at U = 0 V and at the limiting potential (1.26 V vs. SHE) under alkaline conditions (pH = 11). Ti, O, Ni, N, and H atoms are represented by light blue, red, gray, blue, and white spheres, respectively.


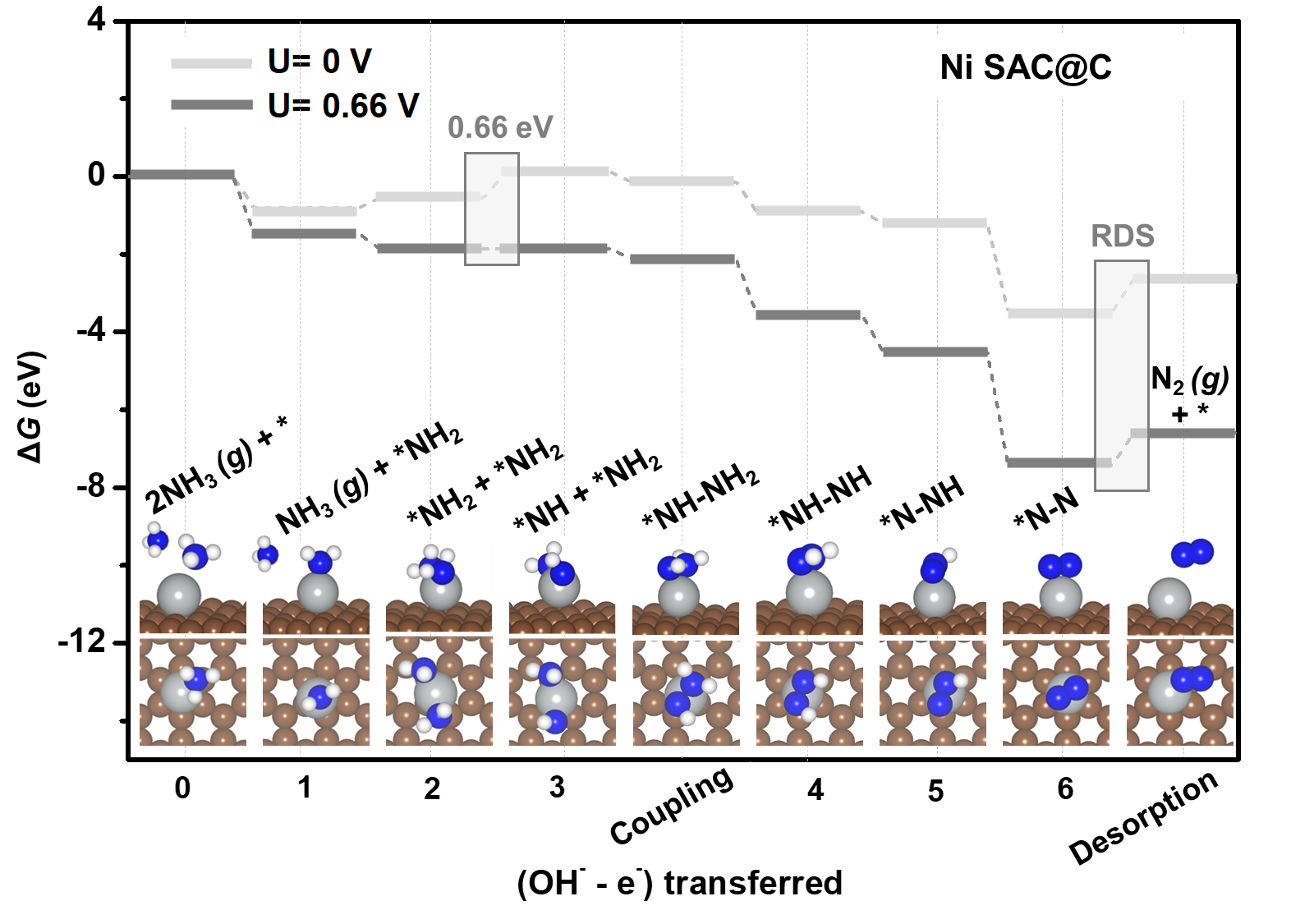


**Figure S41.** Calculated free-energy diagram for the AOR via the G-M pathway toward N_2_ formation on the Ni SAC@C surface at U = 0 V and at the limiting potential (0.66 V vs. SHE) under alkaline conditions (pH = 11). C, Ni, N, and H atoms are represented by brown, gray, blue, and white spheres, respectively.

**
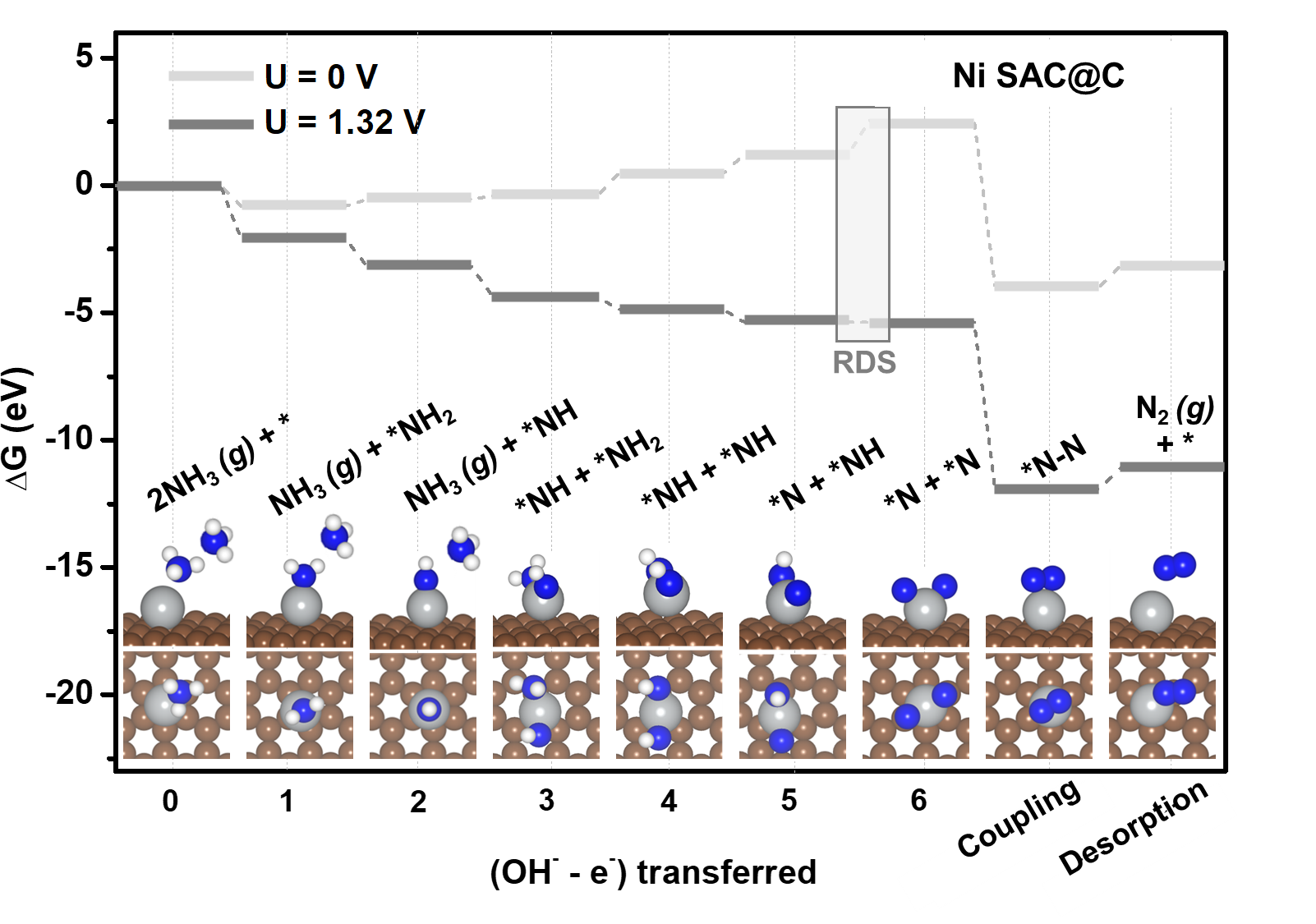
**

**Figure S42.** Calculated free-energy diagram for the AOR via the O-S pathway toward N_2_ formation on the Ni SAC@C surface at U = 0 V and at the limiting potential (1.32 V vs. SHE) under alkaline conditions (pH = 11). C, Ni, N, and H atoms are represented by brown, gray, blue, and white spheres, respectively.

**
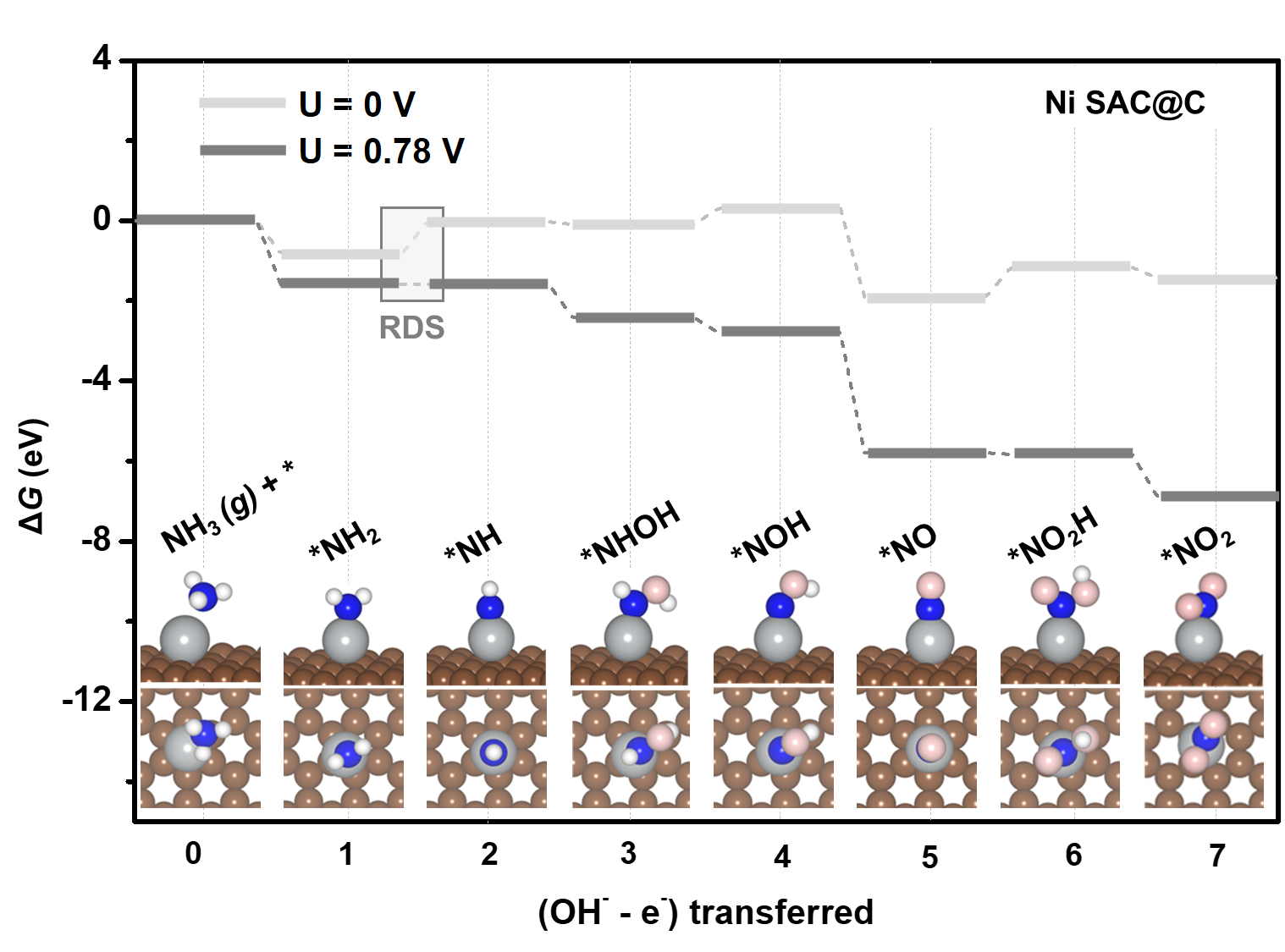
**

**Figure S43.** Calculated free-energy diagram for the AOR toward NO_2_ formation on the Ni SAC@C surface at U = 0 V and at the limiting potential (0.78 V vs. SHE) under alkaline conditions (pH = 11). C, Ni, N, O and H atoms are represented by brown, gray, blue, light pink and white spheres, respectively.


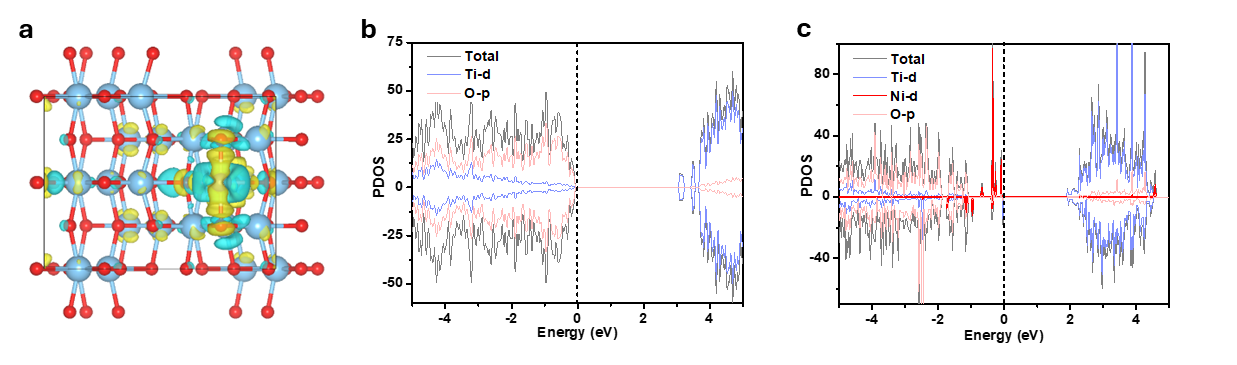


**Figure S44.** (a) Top view of the charge density difference of Ni SAC@TiO_2_. Electron accumulation and depletion are indicated by yellow and blue regions, respectively. The isovalue is set to 0.002 *e*/bohr^3^. Ti, and O atoms are represented by light blue and red spheres, respectively. Projected density of state (PDOS) of (b) pristine TiO_2_ and (c) Ni SAC@TiO_2_.

**Table S1.** ICP-AES spectroscopy results.

| **Sl No.** | **Materials** | **Wt% of Metal** |
| --- | --- | --- |
| 1. | Ni SAC@TiO_2_ | 0.302% |
| 2. | Cu SAC@TiO_2_ | 0.310% |
| 3. | Co SAC@TiO_2_ | 0.305% |
| 4. | Ni SAC@C | 0.214% |
| 5. | Ni NP@TiO_2_ | 9.948% |

**Table S2.** Structural parameters of Ni SAC@TiO_2_ and Ni SAC@C samples extracted from the Ti K-edge EXAFS fitting curves.

| **Sample** | **Scattering**  **Shell** | **CN** | **R(Å)** | **σ^2^ (Å^2^)** | **R factor** |
| --- | --- | --- | --- | --- | --- |
| Ni SAC@TiO_2_ | Ti-O | 3 | 1.52 | 0.0028 | 0.01 |
| TiO_2_ | Ti-O | 3 | 1.57 | 0.0025 | 0.01 |

**Table S3.** Structural parameters of Ni SAC@TiO_2_ and TiO_2_ samples extracted from the Ni K-edge EXAFS fitting curves.

| **Sample** | **Scattering**  **Shell** | **CN** | **R(Å)** | **σ^2^ (Å^2^)** | **R factor** |
| --- | --- | --- | --- | --- | --- |
| Ni SAC@TiO_2_ | Ni-O | 4 | 1.35 | 0.0112 | 0.02 |
| Ni SAC@C | Ni-C | 6 | 2.21 | 0.0069 | 0.02 |

CN is the coordination number; R is interatomic distance (the bond length between central atoms and surrounding coordination atoms); σ^2^ is Debye-Waller factor (a measure of thermal and static disorder in absorber-scatterer distances); R factor is used to value the goodness of the fitting.

**Table S4.** Faradaic efficiency calculation

| **Catalyst** | **% FE of N_2_** | **% FE of O_2_** | **% FE of NO_3_^-^** |
| --- | --- | --- | --- |
| TiO_2_ | 2.98 | 6.22 | 38.20 |
| Ni SAC@TiO_2_ | 35.12 | 3.85 | 16.66 |
| Cu SAC@TiO_2_ | 2.17 | 7.46 | 36.00 |
| Co SAC@TiO_2_ | 3.85 | 6.41 | 35.12 |

**Table S5.** Comparison typical non-noble based electrocatalysts of AOR with Ni SAC@TiO_2_.

| **Catalysts** | **Electrolyte** | **Onset potential**  **(V vs RHE)** | **Current density**  **(mA cm^-2^) at 1.45 V vs RHE** | **Scan rate**  **(mV s^-1^)** | **Ref.** |
| --- | --- | --- | --- | --- | --- |
| Vo-rich CuO | 1.0 M KOH + 1.0 M NH_3_ | ≈1.36 | 60 | 10 | 1 |
| Ni_0.8_Cu_0.2_ LHs | 0.5 M NaOH + 55 mM NH_4_Cl | ≈1.44 | 5 | 25 | 2 |
| Ni_4_Cu_1_Co_1.5_-BP | 1 M KOH + 0.5 M NH_3_ | ≈1.38 | 52 | 50 | 3 |
| Ni_1_Cu_3_-S-T/CP | 1 M NaOH + 0.2 M NH_4_Cl | ≈1.40 | 20 | 20 | 4 |
| Ni_1_Cu_1_Co_0.5_-S-T/CP | 1 M NaOH + 0.2 M NH_4_Cl | ≈1.37 | 30 | 20 | 5 |
| Ni(OH)_2__Cu_2_O@CuO | 1 M KOH + 1 M NH_3_ | ≈1.39 | 20 | 10 | 6 |
| Ni_10_Cu_1_/BDD | 0.5 M NaOH + 0.5 M NH_3_-N | ≈1.35 | ＜5 | 50 | 7 |
| Ni_0.8_Cu_0.2_ | 0.1 M KOH+ 0.01M (NH_4_)_2_SO_4_ | ≈1.42 | ＜5 | 10 | 8 |
| Ni_4_Cu_5_Fe1-C | 0.5 M KOH + 55 mM NH_4_Cl | ≈1.44 | ＜5 | 5 | 9 |
| NiCuCo-CNT | 1 M KOH+ 0.5 M NH_3_ | ≈1.38 | 18 | 25 | 10 |
| Ni SAC@TiO_2_ | 0.1 M NaClO_4_ + 0.1 M NH_3_ | ≈1.57 | ＜5 | 10 | Our Work |

**Table S6.** Calculated total energies (*E*), zero-point energy corrections (*ZPE*), entropy contributions (*T*Δ*S*), and Gibbs free energies (*G*) of gaseous molecules at 298.15 K.

| **Species** | ***E* (eV)** | ***ZPE* (eV)** | ***T***Δ***S* (eV)** | ***G* (eV)** |
| --- | --- | --- | --- | --- |
| H_2_O | -14.22 | 0.56 | 0.58 | -14.25 |
| H_2_ | -6.78 | 0.26 | 0.40 | -6.92 |
| NH_3_ | -19.54 | 0.58 | 0.60 | -19.55 |

**Table S7.** Calculated total energies (*E*), zero-point energy corrections (*ZPE*), entropy contributions (*T*Δ*S*), and Gibbs free energies (*G*) of surface-bound intermediates involved in the AOR on the TiO_2_ catalyst at 298.15 K.

| **Species** | ***E* (eV)** | ***ZPE* (eV)** | ***T***Δ***S* (eV)** | ***G* (eV)** |
| --- | --- | --- | --- | --- |
| *NH_2_ | -651.44 | 0.70 | 0.15 | -650.89 |
| *NH | -645.62 | 0.36 | 0.13 | -645.39 |
| *N | -640.13 | 0.08 | 0.15 | -640.20 |
| *NH_2_ + *NH_2_ | -666.01 | 1.42 | 0.26 | -664.85 |
| *NH + *NH_2_ | -660.23 | 1.07 | 0.25 | -659.41 |
| *NH + *NH | -654.42 | 0.94 | 0.33 | -653.81 |
| *N + *NH | -648.93 | 0.49 | 0.13 | -648.57 |
| *N + *N | -644.74 | 0.19 | 0.18 | -644.73 |
| *N-N (coupling) | -653.77 | 0.28 | 0.23 | -653.72 |
| *NO | -649.54 | 0.25 | 0.24 | -649.53 |
| *NOH | -653.63 | 0.43 | 0.18 | -653.38 |
| *NHOH | -657.34 | 0.87 | 0.16 | -656.63 |
| *NH_2_OH | -662.64 | 1.25 | 0.19 | -661.58 |
| *HNO_2_ | -660.41 | 0.70 | 0.25 | -659.96 |
| *NO_2_ | -655.36 | 0.39 | 0.41 | -655.38 |

**Table S8.** Calculated total energies (*E*), zero-point energy corrections (*ZPE*), entropy contributions (*T*Δ*S*), and Gibbs free energies (*G*) of surface-bound intermediates involved in the AOR on the Ni SAC@TiO_2_ catalyst at 298.15 K.

| **Species** | ***E* (eV)** | ***ZPE* (eV)** | ***T***Δ***S* (eV)** | ***G* (eV)** |
| --- | --- | --- | --- | --- |
| *NH_2_ | -657.00 | 0.74 | 0.11 | -656.37 |
| *NH | -651.59 | 0.38 | 0.12 | -651.33 |
| *N | -647.35 | 0.11 | 0.04 | -647.28 |
| *NH_2_ + *NH_2_ | -673.38 | 1.47 | 0.24 | -672.15 |
| *NH_2_-NH_2_ (coupling) | -672.83 | 1.62 | 0.15 | -671.36 |
| *NH + *NH_2_ | -668.27 | 1.14 | 0.20 | -667.33 |
| *NH-NH_2_ (coupling) | -668.57 | 1.24 | 0.12 | -667.45 |
| *NH + *NH | -662.58 | 0.77 | 0.18 | -661.99 |
| *NH-NH (coupling) | -664.79 | 0.91 | 0.12 | -664.00 |
| *N + *NH | -657.99 | 0.51 | 0.14 | -657.62 |
| *N-NH (coupling) | -661.04 | 0.63 | 0.18 | -660.59 |
| *N + *N | -652.30 | 0.20 | 0.15 | -652.25 |
| *N-N (coupling) | -658.00 | 0.27 | 0.23 | -657.96 |
| *NO | -655.73 | 0.27 | 0.17 | -655.63 |
| *NOH | -658.29 | 0.54 | 0.11 | -657.86 |
| *NHOH | -662.71 | 0.88 | 0.16 | -661.99 |
| *NH_2_OH | -666.67 | 1.24 | 0.26 | -665.69 |
| *HNO_2_ | -665.18 | 0.73 | 0.26 | -664.71 |
| *NO_2_ | -661.56 | 0.33 | 0.09 | -661.32 |

**Table S9.** Calculated total energies (*E*), zero-point energy corrections (*ZPE*), entropy contributions (*T*Δ*S*), and Gibbs free energies (*G*) of surface-bound intermediates involved in the AOR on the Ni SAC@C catalyst at 298.15 K.

| **Species** | ***E* (eV)** | ***ZPE* (eV)** | ***T***Δ***S* (eV)** | ***G* (eV)** |
| --- | --- | --- | --- | --- |
| *NH_2_ | -315.33 | 0.72 | 0.18 | -314.79 |
| *NH | -310.14 | 0.38 | 0.13 | -309.89 |
| *N | -304.76 | 0.10 | 0.12 | -304.78 |
| *NH_2_ + *NH_2_ | -331.14 | 1.47 | 0.29 | -329.96 |
| *NH + *NH_2_ | -326.04 | 1.03 | 0.19 | -325.20 |
| *NH-NH_2_ (coupling) | -326.44 | 1.14 | 0.15 | -325.45 |
| *NH + *NH | -320.74 | 0.77 | 0.28 | -320.25 |
| *NH-NH (coupling) | -322.78 | 0.81 | 0.13 | -322.10 |
| *N + *NH | -315.60 | 0.36 | 0.11 | -315.35 |
| *N-NH (coupling) | -318.65 | 0.68 | 0.32 | -318.29 |
| *N + *N | -309.90 | 0.19 | 0.21 | -309.92 |
| *N-N (coupling) | -316.53 | 0.27 | 0.19 | -316.45 |
| *NO | -313.78 | 0.27 | 0.17 | -313.68 |
| *NOH | -315.88 | 0.54 | 0.26 | -315.60 |
| *NHOH | -320.69 | 0.88 | 0.28 | -320.09 |
| *NH_2_OH | -324.77 | 1.23 | 0.29 | -323.83 |
| *HNO_2_ | -323.43 | 0.69 | 0.34 | -323.08 |
| *NO_2_ | -319.32 | 0.29 | 0.20 | -319.23 |

**References**

1. Huang, J., Chen, Z., Cai, J., Jin, Y., Wang, T., and Wang, J. (2022). Activating copper oxide for stable electrocatalytic ammonia oxidation reaction via in-situ introducing oxygen vacancies. Nano Res. *15*, 5987-5994.

2. Xu, W., Lan, R., Du, D., Humphreys, J., Walker, M., Wu, Z., Wang, H., and Tao, S. (2017). Directly growing hierarchical nickel-copper hydroxide nanowires on carbon fibre cloth for efficient electrooxidation of ammonia. Appl. Catal. B: Environ. *218*, 470-479.

3. Hu, Z., Lu, S., Tang, F., Yang, D., Zhang, C., Xiao, Q., and Ming, P. (2023). High-performance precious metal-free direct ammonia fuel cells endowed by Co-doped Ni4Cu1 anode catalysts. Appl. Catal. B: Environ. *334*, 122856.

4. Zhang, H., Wang, H., Tong, X., Zhou, L., Yang, X., Wang, Y., Zhang, M., and Wu, Z. (2023). Sulfur induced surface reconfiguration of Ni1Cu3-S-T/CP anode for high-efficiency ammonia electro-oxidation. Chem. Eng. J. *452*, 139582.

5. Wang, H., Tong, X., Zhou, L., Wang, Y., Liao, L., Ouyang, S., and Zhang, H. (2022). Unique three-dimensional nanoflower-like NiCu electrodes constructed by Co, S co-doping for efficient ammonia oxidation reaction. Sep. Purif. Technol. *303*, 122293.

6. Huang, J., Cai, J., and Wang, J. (2020). Nanostructured wire-in-plate electrocatalyst for high-durability production of hydrogen and nitrogen from alkaline ammonia solution. ACS Appl. Energy Mater. *3*, 4108-4113.

7. Song, J., Yinhai, Y., Jia, Y., Wang, T., Wei, J., Wang, M., Zhou, S., Li, Z., Hou, Y., Lei, L., et al. (2021). Improved NH3-N conversion efficiency to N2 activated by BDD substrate on NiCu electrocatalysis process. Sep. Purif. Technol. *276*, 119350.

8. Jiang, X., Ying, D., Liu, X., Liu, M., Zhou, S., Guo, C., Zhao, G., Wang, Y., and Jia, J. (2020). Identification of the role of Cu site in Ni-Cu hydroxide for robust and high selective electrochemical ammonia oxidation to nitrite. Electrochim. Acta *345*, 136157.

9. Zhang, M., Zhang, J., Jeerh, G., Zou, P., Sun, B., Walker, M., Xie, K., and Tao, S. (2022). A symmetric direct ammonia fuel cell using ternary NiCuFe alloy embedded in a carbon network as electrodes. J. Mater. Chem. A *10*, 18701-18713.

10. Liu, Z., Wu, M., and Ma, J. (2022). Ni–Cu–Co grid-like hydroxyl oxide ammonia oxidation reaction catalyst supported on carbon nanotubes. Energy Fuels *36*, 10339-10345.
